# Parent-Targeted Oral Health Text Messaging for Underserved Children Attending Pediatric Clinics: A Randomized Clinical Trial

**DOI:** 10.1001/jamanetworkopen.2024.52780

**Published:** 2025-01-02

**Authors:** Belinda Borrelli, Romano Endrighi, Timothy Heeren, William G. Adams, Stuart A. Gansky, Scott Werntz, Nicolle Rueras, Danielle Stephens, Niloufar Ameli, Michelle M. Henshaw

**Affiliations:** 1 Center for Behavioral Science Research, Boston University Henry M. Goldman School of Dental Medicine, Boston, Massachusetts; 2 Department of Biostatistics, Boston University School of Public Health, Boston, Massachusetts; 3 Department of Pediatrics, Boston Medical Center and Boston University Chobanian and Avedisian School of Medicine, Boston, Massachusetts; 4 Center to Address Disparities in Children’s Oral Health, University of California, San Francisco School of Dentistry; 5 Bakar Computational Health Sciences Institute, University of California, San Francisco; 6 Accrete Health Partners LLC, Lincolnshire, Illinois; 7 Boston College School of Social Work, Boston, Massachusetts; 8 Academic Research Services, University of California, San Francisco; 9 Department of Preventive and Restorative Dental Sciences, University of California, San Francisco School of Dentistry; 10 Office of Global and Population Health, Boston University Henry M. Goldman School of Dental Medicine, Boston, Massachusetts

## Abstract

**Importance:**

Caries is the most common chronic childhood disease, with substantial health disparities.

**Objective:**

To test whether parent-targeted oral health text (OHT) messages outperform child wellness text (CWT) messages on pediatric caries increment and oral health behaviors among underserved children attending pediatric well-child visits.

**Design, Setting, and Participants:**

The parallel randomized clinical trial, Interactive Parent-Targeted Text Messaging in Pediatric Clinics to Reduce Caries Among Urban Children (iSmile), included participants who were recruited during pediatric medical clinic visits at 4 sites in Boston, Massachusetts, that serve low-income and racially and ethnically diverse (herein, underserved) populations. English-speaking or Spanish-speaking caregivers of children younger than 7 years with at least 1 tooth were eligible. The study, which had a dose-matched design, was conducted from March 9, 2018, to February 28, 2022, with a 24-month follow-up. Text messages were sent for 4 months, plus a 1-month booster at 12 months.

**Intervention:**

Text messages were bilingual, automated, interactive, customized, and gamified. OHT messages focused on child toothbrushing and preventive dental visits. CWT messages focused on reading and child safety. Both included a choice of other content topics pertinent to their randomized arm.

**Main Outcomes and Measures:**

The primary outcome was 24-month caries increment assessed by calibrated examiners. Secondary outcomes were oral health behaviors known to predict pediatric caries (eg, toothbrushing, sugar-sweetened beverages, diet, fluoride toothpaste use, and preventive dental visits), which were assessed by self-report, and participant satisfaction with the text message program. Caries examinations occurred at baseline and 12 and 24 months later. Self-report surveys occurred at these time points and at the end of the text message program (4 months).

**Results:**

Among 1388 caregivers who were approached for eligibility, 969 were eligible. Of these, 754 caregivers (mean [SD] age, 32.9 [7.2] years; 713 female [94.6%]) and 754 children (mean [SD] age, 2.9 [1.7] years; 377 female [50.0%]) were randomized (77.8%); 449 of 657 participants (68.3%) were below the poverty line. Responses to text messages were high (OHT group: 67.9%; CWT group: 69.6%). There were no significant group differences in caries increment (OHT group: 43.0% vs CWT group: 42.7%; adjusted odds ratio, 0.99 [95% CI, 0.63-1.56]). Children in the OHT messaging group were significantly more likely to meet toothbrushing guidelines (odds ratio [OR], 1.77 [95% CI, 1.13-2.78]), have preventive dental visits (pooled OR, 1.51 [95% CI, 1.18-1.94]), and use fluoride toothpaste (pooled OR, 1.46 [95% CI, 1.06-2.01]) compared with those in the CWT messaging group over 24 months. OHT messages had a significant effect on caregivers’ own toothbrushing (pooled mean difference, 0.48 [95% CI, 0.03-0.92]).

**Conclusion and Relevance:**

The findings of this randomized clinical trial including underserved children and their caregivers suggest that OHT messages had significant and sustained effects on preventive dental behaviors known to reduce caries among both caregivers and children, but had no effect on caries increment in children. Highly appealing and low-burden text messages coupled with evidence of change in oral health behaviors may be important in reducing oral health disparities.

**Trial Registration:**

ClinicalTrials.gov Identifier: NCT03294590

## Key Points

**Question:**

Can a bilingual, parent-targeted text messaging program improve oral health behaviors and decrease caries increment in a low-income and racially and ethnically diverse population of children?

**Findings:**

In this randomized clinical trial of 754 parent and child dyads, oral health text messages (vs child wellness text messages) had no effect on child caries increment. The intervention was effective for secondary outcomes of child toothbrushing, fluoride use, preventive dental visits, and caregiver toothbrushing.

**Meaning:**

The findings of this study suggest that providing a low-burden text-messaging intervention for a low-income and racially and ethnically diverse sample of pediatric patients and their caregivers improved oral health behaviors in this population.

## Teaser

This randomized clinical trial assesses whether oral health text messages outperform child wellness text messages on pediatric caries increment among underserved children and their caregivers.

## Introduction

Although there are effective preventive treatments for dental caries, caries is the most common chronic childhood disease, with associated racial, ethnic, and income oral health inequities.^1,2^ Over 90% of US children attend well-child primary care visits, and the American Academy of Pediatrics identified these visits as important to reach children at high risk for caries.^3,4^ However, time constraints often prevent pediatricians from counseling parents to improve their children’s oral health. Because over 95% of adults in the US regularly use text messaging, with no disparities in race, ethnicity, or income,^5,6^ text messages can reach populations at highest risk for caries with ongoing and tailored behavior change messages. To our knowledge, extant text messaging studies to improve pediatric oral health have small samples and short-term outcomes,^7,8,9^ and they lack rigorous controls or do not describe them.^7,8,9,10^

In response to this gap, we previously conducted a series of studies to develop an interactive and gamified parent-targeted text message intervention to reduce caries among a low-income and racially and ethnically diverse (herein, underserved) population of families visiting urban pediatric medical clinics. Codesigning the program with the target population (focus groups) and pilot testing^11^ helped ensure that the program addressed health literacy and cultural considerations, as well as theoretical foundations^12^ and clinical guidelines.^4,13^ The current trial (Interactive Parent-Targeted Text Messaging in Pediatric Clinics to Reduce Caries Among Urban Children [iSmile]) extends the pilot by recruiting a larger sample, measuring caries objectively, partnering with multiple pediatric clinics to increase generalizability, lengthening the intervention and follow-up period, and using novel text messaging engagement strategies and features. We hypothesized that children of caregivers randomized to oral health text (OHT) messages would have a lower 24-month caries increment compared with children of caregivers randomized to a comparison control group, child wellness text (CWT) messages. We also hypothesized that OHT messages would improve oral health behaviors that have been shown to predict caries, such as child toothbrushing, preventive dental visits, fluoride use, healthy eating, and reduced sugar-sweetened beverages, and that caregivers receiving OHT messages would engage in more toothbrushing than those receiving CWT messages.

## Methods

### Participants

Eligible participants in this randomized clinical trial were parents (or caregivers) of children younger than 7 years (with ≥1 tooth) who were pediatric patients at 1 of 3 community health centers or 1 safety-net hospital in Boston, Massachusetts. Recruitment occurred by clinic staff referral (including W.G.A.) or by research assistants (including N.R.) in pediatric waiting rooms (from March 2018 to March 2020). Research assistants obtained written informed consent. Consenting participants completed a baseline questionnaire, and their child received an oral health assessment (OHA) before randomization to either the OHT or CWT group. All materials, including text messages, were translated into Spanish. The study and trial protocol (Supplement 1) were approved by the Boston University Medical Campus Institutional Review Board. Data collection began on March 9, 2018, and ended on February 28, 2022. This study followed the Consolidated Standards of Reporting Trials (CONSORT) reporting guideline for randomized clinical trials.

Participants were ineligible if they were younger than 18 years, did not understand and read English or Spanish^14^ or own a mobile telephone, participated in our pilot studies, had a diagnosis of a serious mental illness or a substance use disorder, had a child with severe congenital tooth malformations, were currently in another text message or oral health study, planned on moving within 2 years or leaving the country for 2 months or more, or their child did not complete the OHA.

### Randomization

A permuted randomized block design was used to randomize participants to the OHT or CWT group (random-sized blocks of 2 or 4 participants), stratified by study site and history of caries (no caries, any caries). The National Institute of Dental and Craniofacial Research, National Institutes of Health–appointed data coordinating center created the randomization schedule using SAS statistical software, version 9.4 (SAS Institute Inc) and administered it using the REDCap database. None of the authors collected outcome data. Only S.A.G., N.R., and N.A. were not masked to the randomization condition; the rest of the authors were masked to randomization condition.

### Text Message Programs

Both OHT and CWT groups received bilingual text messages for 4 months, followed by a 1-month booster session occurring 8 months later (see sample text messages in the eMethods in Supplement 2). Both groups were matched on program structure and features (duration, receipt of text messages twice per day for the first month and then once per day, engagement strategies, behavioral goal-shaping, and automated interactivity).

The OHT group’s core text message content included modules on brushing (eg, technique, challenges, and frequency) and visiting the dentist (eg, age to schedule first appointment, finding a dentist, and what to expect at dental visits). Caregivers were also able to choose other text message oral health topics (bedtime routine, bottle or sippy-cup use, sugar-sweetened beverages, healthy eating, getting fluoride, and fun facts). The CWT group’s core text message content included modules on reading and child safety, and caregiver choice topics were physical activity, healthy development, secondhand smoke, safety hazards, sleep behavior, and stress.

Text message engagement strategies for both groups included (1) photographs, video, and quizzes; (2) topic choice; (3) interactive yet automated text messages; (4) customization and personalization; and (5) electronic badges rewarding successive approximations of desired behaviors (behavioral shaping). For the OHT group, earning badges unlocked higher-level characters (supertooth heroes: Molly Molar, Charlie Chew, Faye Fluoride, and Captain Chomp) by achieving progressively greater brushing goals, with a goal of brushing twice per day, every day. A map showed progress, and bonuses could be unlocked (Toothtastic Rock Stars). The reward structure was similar for the CWT group but consisted of different badges and characters (Book Buddies).

### Measures

Self-report assessments occurred electronically at baseline and at the end of the program (4 months), immediately before the 1-month booster (12 months), and 24 months after baseline. Pediatric OHAs were conducted in person at baseline and at 12 and 24 months by calibrated clinical examiners at the clinic in which the child received care. Participants could earn a total of $330 for completing surveys and OHAs. Self-reported sociodemographics (age, sex, number of children, race and ethnicity, income, educational level, marital status, and employment) were obtained at baseline, with response options defined by 2 researchers (B.B. and M.M.H.). Race and ethnicity categories included American Indian or Alaska Native, Asian, Black or African American, Hispanic, Native Hawaiian or Other Pacific Islander, White, more than 1 race, or unknown or not reported. These data were collected because caries prevalence varies by race and ethnicity.

#### Primary Outcome

New surface-level caries was defined as primary tooth surfaces with a cavity at the 24-month OHA and no cavity at baseline. For primary teeth that were present at 12 months but were missing, not due to caries, at 24 months, the tooth surface status at 12 months was used. The data coordinating center conducted annual caries calibration for the project’s dental hygienists.^15^ A κ statistic of at least 0.70 was considered “calibrated.” κ Statistics ranged from 0.85 to 0.90 for in-person calibration sessions. A modified International Caries Detection and Assessment System surface scoring system was used, ranging from 0 to 6, in which 0 to 2 indicates sound to distinct visual change in enamel with no cavitation including white-spot lesions, 3 to 4 indicates localized enamel breakdown with no dentin showing or gray dentin shadow with or without visible cavitation, and 5 to 6 indicates distinct or extensive cavity with visible dentin.^16^ Fillings, sealants, and unerupted surfaces were also documented. OHA sessions were audio recorded, and 20% were reviewed to document that no education occurred.

#### Secondary Outcomes

##### Oral Health Behaviors

Toothbrushing was measured using a valid and reliable timeline follow-back method,^17,18^ in which caregivers visually reviewed the days of the previous week and indicated whether they (or another adult) helped (or supervised) brushing their child’s teeth and the number of times teeth were brushed on those days to yield the total number of child brushings per week (range, 0-21). Adherence to toothbrushing guidelines (twice per day, every day) was then calculated (yes or no). Parents reported their own brushing behavior in the same manner. Fluoride toothpaste use was assessed with a 1-item measure regarding whether fluoride toothpaste was usually used when brushing the child’s teeth (yes or no).^19^ Preventive dental visits were assessed at baseline and at 24 months. Caregivers were asked if their child had been to a dental clinic for a routine checkup or cleaning in the past 12 months (yes or no). At the end of the main text message program (4 months), participants reported this information since baseline and at the 12-month follow-up since the 4-month assessment. Dietary consumption was measured with the 30-item food frequency questionnaire,^20,21^ which has 5 categories of cariogenic risk that were each summed using a weighted formula and then divided by the total to yield an estimated cariogenicity score; greater scores reflect increased consumption of cariogenic food and beverages.^21,22^ Sugar-sweetened beverage consumption was assessed with the 15-item beverage-intake questionnaire for preschool-aged children.^23,24^ The average daily sugar-sweetened beverage consumption (sugar-added beverages and 100% fruit juices) was computed as the product of frequency and amount consumed,^25^ and a median split was used in analyses (higher sugar-sweetened beverage consumption, >4.5 oz; lower, ≤4.5 oz).^24,26^

##### Program Satisfaction

We assessed program length (too long, too short, or about right), design (9 items; 1-7 scale each, with higher scores indicating higher satisfaction) and content (5 items; 1-7 scale each, with higher scores indicating higher satisfaction), and diffusion (showing any text messages to others and the number of people). Participants rated the text message program using 1 to 5 stars, in which 1 indicates one of the worst programs and 5 indicates one of the best programs.

### Sample Size and Power

We initially targeted enrollment of 850 participants (425 in each group), assuming 70% retention (N = 600), and the percent of children with any new caries in primary teeth at 24 months to be 14.3% in the OHT group and 23.9% in the CWT group (providing 84% power), as estimated by previous studies.^27,28^ COVID-19 impacted OHA completion, particularly for the primary outcome of 24-month OHA. The achieved sample size provides 58% power for detecting planned differences in the percent of children with any new primary caries.

### Statistical Analysis

Logistic regression was used to compare the OHT and CWT groups on the odds of a child developing any new caries in primary teeth at 24 months, and proportional odds logistic regression was used to compare the groups on the number of primary teeth with new caries (grouped as 0, 1, 2, 3-5, and ≥6). Mixed-effects logistic regression was used in per-tooth surface analyses to compare the groups on the odds of new caries per primary tooth surface, accounting for clustering of tooth surfaces within a child through random effects. All of these analyses controlled for the presence of any caries at baseline and child age.

The OHT and CWT groups were compared on oral health behaviors assessed at 4, 12, and 24 months through mixed-effects linear regression (for continuous outcomes) and logistic regression (for dichotomous outcomes) models for longitudinal data, controlling for the baseline value of the outcome variable and child age. Interaction terms between time and treatment group were used to estimate separate OHT effects at 4, 12, and 24 months; where the time-by-intervention interaction was not significant, a pooled main effect of intervention across all 3 time points was also estimated. We report odds ratios (ORs) or mean differences as appropriate with 95% CIs, and 2-sided *P* ≤ .05 was considered statistically significant.

A substantial number of children did not complete the 24-month OHA, largely due to the COVID-19 epidemic and the necessity for the OHA to be conducted in person. Our study protocol specified that if data were available for less than 70% of participants, primary analyses would be conducted using multiple imputation (eAppendix in Supplement 2). Preliminary analyses examined univariate associations between baseline variables and new caries in primary teeth at 24 months. Variables associated with new caries at *P* < .15, along with baseline child age and sex, were used to impute any new caries at 24 months separately for each treatment group. Fifty imputed datasets were generated using the fully conditional specification method in the SAS Multiple Imputation, version 9.4 procedure. Logistic regression controlling for any caries at baseline and child age was used to compare OHT with CWT groups on this outcome.

## Results

Participants included 754 racially and ethnically diverse caregivers (mean [SD] age, 32.9 [7.2] years; 713 female [94.6%] and 41 male [5.4%]) and their children (mean [SD] age, 2.9 [1.7] years; 377 female [50.0%] and 377 male [50.0%]) who were randomized to either the OHT group (n = 377) or the CWT group (n = 377) (Table 1). Of 657 participants, 449 (68.3%) were below the poverty line. For the race and ethnicity categories, 3 caregivers (0.4%) and 3 children (0.4%) were American Indian or Alaska Native, 16 caregivers (2.1%) and 10 children (1.3%) were Asian, 440 caregivers (58.4%) and 424 children (56.2%) were Black or African American, 194 caregivers (25.7%) and 227 children (30.1%) were Hispanic, 3 caregivers (0.4%) and 2 children (0.3%) were Native Hawaiian or Other Pacific Islander, 94 caregivers (12.5%) and 74 children (9.8%) were White, 64 caregivers (8.5%) and 130 children (17.2%) were more than 1 race, and 134 caregivers (17.8%) and 111 children (14.7%) were unknown or not reported.

**Table 1.** Baseline Characteristics of Study Participants^a^

Characteristic	Participant group		
	OHT messaging	CWT messaging	All
Sex			
Parent or caregiver			
Female	358/377 (95.0)	355/377 (94.2)	713/754 (94.6)
Male	19/377 (5.0)	22/377 (5.8)	41/754 (5.4)
Child			
Female	186/377 (49.3)	191/377 (50.7)	377/754 (50.0)
Male	191/377 (50.7)	186/377 (49.3)	377/754 (50.0)
Age, mean (SD), y			
Parent or caregiver	32.8 (7.0)	33.0 (7.4)	32.9 (7.2)
Child	2.9 (1.7)	2.8 (1.6)	2.9 (1.7)
Children in household needing care			
1	143/377 (37.9)	153/376 (40.7)	296/753 (39.3)
2	132/377 (35.0)	121/376 (32.2)	253/753 (33.6)
≥3	102/377 (27.1)	102/376 (27.1)	204/753 (27.1)
Below federal poverty line	214/323 (66.3)	235/334 (70.4)	449/657 (68.3)
Educational level of parent or caregiver			
<High school	42/372 (11.3)	47/371 (12.7)	89/743 (12.0)
High school graduate or GED	122/372 (32.8)	124/371 (33.4)	246/743 (33.1)
Some college (no degree)	99/372 (26.6)	96/371 (25.9)	195/743 (26.2)
College degree	64/372 (17.2)	73/371 (19.7)	137/743 (18.4)
≥Graduate degree	45/372 (12.1)	31/371 (8.4)	76/743 (10.2)
Marital status of parent or caregiver			
Married, living together, or engaged	144/360 (40.0)	124/354 (35.0)	268/714 (37.5)
Divorced, separated, or widowed	50/360 (13.9)	49/354 (13.8)	99/714 (13.9)
Never married	166/360 (46.1)	181/354 (51.1)	347/714 (48.6)
Parent or caregiver employed (≥32 h/wk)	115/364 (31.6)	105/365 (28.8)	220/729 (30.2)
Race			
Parent or caregiver			
American Indian or Alaska Native	2/377 (0.5)	1/377 (0.3)	3/754 (0.4)
Asian	7/377 (1.9)	9/377 (2.4)	16/754 (2.1)
Black or African American	235/377 (62.3)	205/377 (54.4)	440/754 (58.4)
Native Hawaiian or Other Pacific Islander	1/377 (0.3)	2/377 (0.5)	3/754 (0.4)
White	46/377 (12.2)	48/377 (12.7)	94/754 (12.5)
More than 1 race	25/377 (6.6)	39/377 (10.3)	64/754 (8.5)
Unknown or not reported	61/377 (16.2)	73/377 (19.4)	134/754 (17.8)
Child			
American Indian or Alaska Native	2/377 (0.5)	1/377 (0.3)	3/754 (0.4)
Asian	6/377 (1.6)	4/377 (1.1)	10/754 (1.3)
Black or African American	225/377 (59.7)	199/377 (52.8)	424/754 (56.2)
Native Hawaiian or Other Pacific Islander	1/377 (0.3)	1/377 (0.3)	2/754 (0.3)
White	33/377 (8.7)	41/377 (10.9)	74/754 (9.8)
More than 1 race	58/377 (15.4)	72/377 (19.1)	130/754 (17.2)
Unknown or not reported	52/377 (13.8)	59/377 (15.6)	111/754 (14.7)
Hispanic ethnicity			
Parent or caregiver	98/377 (26.0)	96/377 (25.5)	194/754 (25.7)
Child	114/377 (30.2)	113/377 (30.0)	227/754 (30.1)

Abbreviations: CWT, child wellness text; GED, General Educational Development; OHT, oral health text.

^a^ Unless noted otherwise, data are presented as the No./total No. (%) of participants. Some characteristics do not include the full participant group because of missing data (ie, the participant selected not to answer).

As shown in the Figure, among 1388 participants who were approached for eligibility, 969 (69.8%) were screened as eligible; of those, 895 caregivers (92.4%) provided informed consent, and 754 completed baseline activities (77.8%) and were randomized. There were no significant group differences in survey completion (4 months: 76.7%; 12 months: 75.9%; and 24 months: 71.9%) or OHA completion (12 months: 51.8% and 24 months: 41.9%). Those who did not complete the 24-month OHA (n = 438) were more likely to be below the poverty level (72.1% vs 63.2%; *P* = .02), have less than a college education (76.6% vs 64.1%; *P* < .001), and not be partnered (65.9% vs 57.8%; *P* = .009). Fifteen participants (7 in the OHT group, 8 in the CWT group) dropped out of the study. Only 47 caregivers (6.2%) opted out of the main text message program (OHT group, 6.1%; CWT group, 6.3%), and 34 of 711 caregivers (4.8%) opted out of the booster session (OHT group, 4.8%; CWT group, 4.8%).

**Figure.**
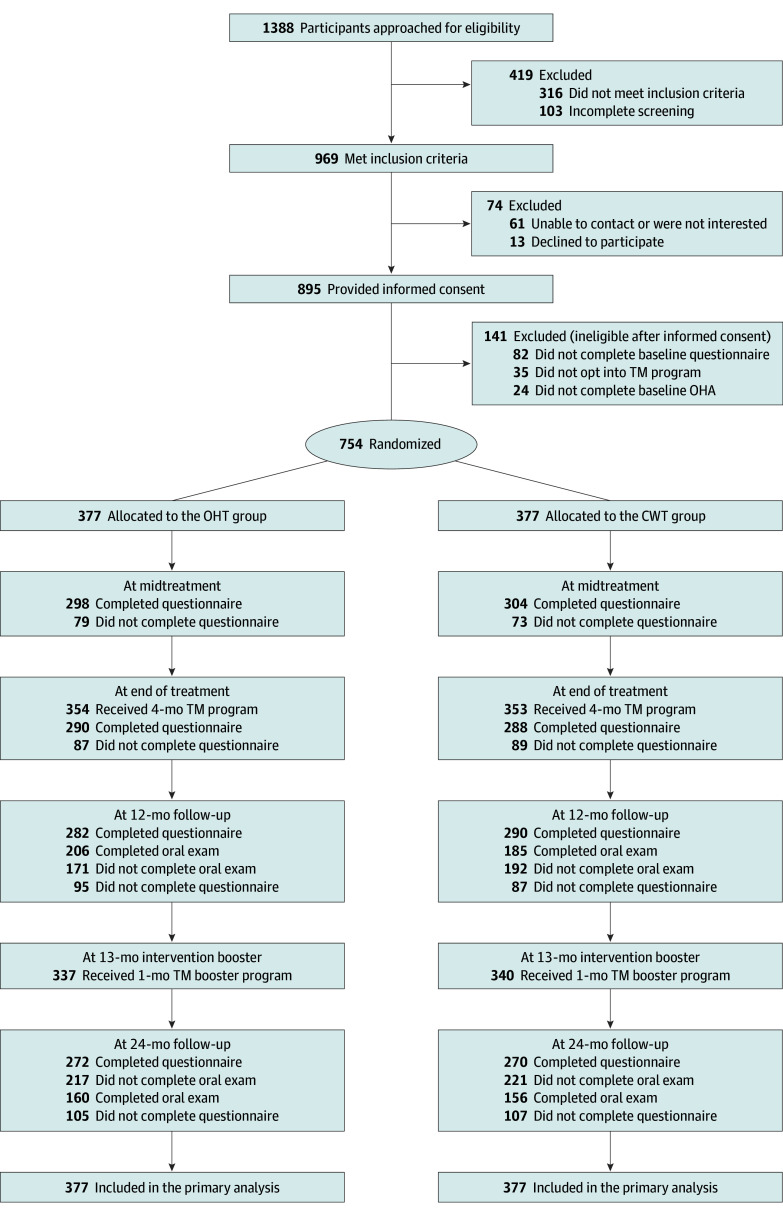
CONSORT Diagram of Participant Caregivers’ Flow Through the Study CWT indicates child wellness text; OHA, oral health assessment; OHT, oral health text; TM, text message.

### Engagement in the Text Message Program and Program Satisfaction

Text message response rates were high (OHT group, 67.9%; CWT group, 69.6%). Participants were highly satisfied with program length and design and content, including cultural and linguistic relevance (Table 2). The mean (SD) satisfaction score was 6.1 (1.3), on a scale ranging from 1 to 7, for the OHT group and 6.0 (1.4) for the CWT group. Most participants rated the program as 4 or 5 stars (OHT group, 80.7%; CWT group, 79.1%).

**Table 2.** Participant Program Satisfaction^a^

Program engagement	Participant group		
	OHT messaging	CWT messaging	All
Did you show the iSmile text messages to anyone else (or forward them)? Yes	168/290 (57.9)	177/288 (61.5)	345/578 (59.7)
No. of people?			
1	23/168 (13.7)	16/177 (9.0)	39/345 (11.3)
2	45/168 (26.8)	46/177 (26.0)	91/345 (26.4)
3	40/168 (23.8)	39/177 (22.0)	79/345 (22.9)
4	17/168 (10.1)	21/177 (11.9)	38/345 (11.0)
5	22/168 (13.1)	26/177 (14.7)	48/345 (13.9)
≥6	21/168 (12.5)	29/177 (16.4)	50/345 (14.5)
You have been receiving the iSmile text messages for about 4 mo. What do you think about the length of the program?			
It was just about right.	193/290 (66.6)	172/288 (59.7)	365/578 (63.1)
The program should last longer.	68/290 (23.4)	80/288 (27.8)	148/578 (25.6)
The program should be shorter.	29/290 (10.0)	36/288 (12.5)	65/578 (11.2)
How much longer should the iSmile texts have lasted, in months?			
1	1/68 (1.5)	3/80 (3.8)	4/148 (2.7)
2	10/68 (14.7)	12/80 (15.0)	22/148 (14.9)
3	5/68 (7.4)	7/80 (8.8)	12/148 (8.1)
4	3/68 (4.4)	9/80 (11.3)	12/148 (8.1)
5	2/68 (2.9)	2/80 (2.5)	4/148 (2.7)
≥6	47/68 (69.1)	47/80 (58.8)	94/148 (63.5)
Satisfaction score, mean (SD)^b^			
Relevant for your cultural background	5.8 (1.6)	5.6 (1.7)	5.7 (1.7)
No. of participants	279	282	561
Age appropriate for your child	6.0 (1.4)	6.0 (1.4)	6.0 (1.4)
No. of participants	281	284	565
Easy to understand the content of the text messages	6.5 (1.1)	6.6 (1.0)	6.5 (1.0)
No. of participants	284	285	569
Easy to integrate into your day and routine	6.2 (1.2)	6.2 (1.3)	6.2 (1.2)
No. of participants	283	282	565
Language appropriate	6.5 (1.1)	6.5 (1.1)	6.5 (1.1)
No. of participants	284	282	566
Relevant for you and your child	6.2 (1.4)	6.2 (1.4)	6.2 (1.4)
No. of participants	281	281	562

Abbreviations: CWT, child wellness text; iSmile, Interactive Parent-Targeted Text Messaging in Pediatric Clinics to Reduce Caries Among Urban Children; OHT, oral health text.

^a^ Unless noted otherwise, data are presented as the No./total No. (%) of participants.

^b^ Scores range from 1 to 7, with higher scores indicating higher satisfaction.

### Caries Increment at the 24-Month Follow-Up

Preliminary analyses of children with a completed 24-month OHA identified several baseline variables for multiple imputation analyses: number of children per caregiver, full-time employment, estimated cariogenicity score, and the presence of any caries as associated with any new caries in primary teeth at 24 months. These variables and child age and sex were used to impute any new caries at 24 months in 50 datasets.

The percent of children having new caries in primary teeth at the 24-month OHA was 43.0% of the OHT group and 42.7% of the CWT group (Table 3), with no significant differences between treatment groups (adjusted OR [AOR], 0.99 [95% CI, 0.63-1.56]). The difference between groups in the number of primary teeth with new caries was not significant (AOR, 1.15 [95% CI, 0.74-1.79]). In surface-level analyses, the percent of surfaces with new caries at the 24-month OHA was 4.02% for the OHT group (n = 545) and 3.68% for the CWT group (n = 496), with no significant differences between groups (AOR, 1.10 [95% CI, 0.67-1.79]).

**Table 3.** New Caries in Primary Teeth at the 24-Month Follow-Up^a^

Variable	Participant group		AOR (95% CI)
	OHT messaging	CWT messaging	
Per-child analysis			
Baseline (any caries)	72/377 (19.1)	68/377 (18.0)	NA
Any new caries in primary teeth at 24 mo (multiple imputation^b^)	162/377 (43.0)	161/377 (42.7)	0.99 (0.63-1.56)
No. of primary teeth with new caries at 24 mo	NA	NA	1.15 (0.74-1.79)
0	91/160 (56.9)	95/156 (60.9)	NA
1	19/160 (11.9)	15/156 (9.6)	NA
2	13/160 (8.1)	16/156 (10.3)	NA
3-5	22/160 (13.7)	16/156 (10.3)	NA
≥6	15/160 (9.4)	14/156 (9.0)	NA
Per-tooth surface analysis			
Noncarious surfaces at baseline, No.	13 553	13 477	NA
Surfaces with new caries at 24 mo, No.	545	496	NA
Surfaces with new caries at 24 mo, %	4.02	3.68	1.10 (0.67-1.79)

Abbreviations: AOR, adjusted odds ratio; CWT, child wellness text; NA, not applicable; OHT, oral health text.

^a^ Unless noted otherwise, data are presented as the No./total No. (%) of participants.

^b^ Data without multiple imputation include OHT: 69 of 160 participants (43.1%); CWT: 61 of 156 participants (39.1%); and AOR (95% CI), 1.13 (0.70-1.80).

### Oral Health Behaviors Through the 24-Month Follow-Up

#### Child Toothbrushing

The OHT group reported more brushings per week during follow-up compared with the CWT group (Table 4). The interaction between OHT messages and time was significant (*P* < .001), indicating that the OHT effect varied over time, with the effects strongest at the 4-month (mean difference, 1.76 [95% CI, 1.07-2.46]) and 24-month (mean difference, 0.91 [95% CI, 0.19-1.62]) follow-up periods. We also examined the treatment effect on toothbrushing guideline adherence. The interaction between OHT messages and time was significant (*P* = .004), with the strongest effects at 4 months (OR, 3.14 [95% CI, 2.00-4.93]) and 24 months (OR, 1.77 [95% CI, 1.13-2.78]).

**Table 4.** Comparison of OHT With CWT Messaging Groups on Oral Health Behaviors

Oral health behavior	Participant group		OHT effect	
	OHT messaging	CWT messaging	Mean difference (95% CI)^a^	OR (95% CI)^b^
**No. of child tooth brushings per wk, mean (SD)**				
Baseline	8.8 (5.8)	8.9 (5.7)	NA	NA
No. of participants	377	377	NA	NA
4 mo	12.9 (3.7)	11.3 (5.0)	1.76 (1.07 to 2.46)	NA
No. of participants	290	288	NA	NA
12 mo	12.0 (4.4)	11.6 (4.6)	0.53 (−0.17 to 1.23)	NA
No. of participants	282	290	NA	NA
24 mo	12.0 (4.5)	11.1 (5.1)	0.91 (0.19 to 1.62)	NA
No. of participants	272	270	NA	NA
Pooled^c^^,^^d^	NA	NA	NA	NA
**Children meeting guidelines for toothbrushing, No./total No. (%)**				
Baseline	144/377 (38.2)	140/377 (37.1)	NA	NA
4 mo	214/290 (73.8)	156/288 (54.2)	NA	3.14 (2.00 to 4.93)
12 mo	167/282 (59.2)	160/290 (55.2)	NA	1.21 (0.79 to 1.87)
24 mo	177/272 (65.1)	147/270 (54.4)	NA	1.77 (1.13 to 2.78)
Pooled^c^^,^^d^	NA	NA	NA	NA
**Children having preventive dental visit, No./total No. (%)**				
Baseline	187/376 (49.7)	185/375 (49.3)	NA	NA
4 mo	162/290 (55.9)	131/288 (45.5)	NA	1.52 (1.05 to 2.22)
12 mo	180/282 (63.8)	157/290 (54.1)	NA	1.47 (1.00 to 2.14)
24 mo	207/272 (76.1)	181/270 (67.0)	NA	1.55 (1.02 to 2.36)
Pooled^c^	NA	NA	NA	1.51 (1.18 to 1.94)
**Children using fluoride, No./total No. (%)**				
Baseline	195/374 (52.1)	200/374 (53.5)	NA	NA
4 mo	225/290 (77.6)	210/288 (72.9)	NA	1.32 (0.83 to 2.09)
12 mo	229/282 (81.2)	215/290 (74.1)	NA	1.62 (1.00 to 2.63)
24 mo	225/272 (82.7)	207/270 (76.7)	NA	1.47 (0.89 to 2.44)
Pooled^c^	NA	NA	NA	1.46 (1.06 to 2.01)
**Higher SSB and fruit juice consumption in children, No./total No. (%)** ^e^				
Baseline	186/373 (49.9)	188/375 (50.1)	NA	NA
4 mo	128/290 (44.3)	141/288 (49.0)	NA	0.81 (0.54 to 1.21)
12 mo	145/282 (51.3)	131/290 (45.0)	NA	1.35 (0.90 to 2.01)
24 mo	125/272 (46.0)	142/270 (52.7)	NA	0.73 (0.49 to 1.11)
Pooled^c^	NA	NA	NA	0.93 (0.72 to 1.21)
**Cariogenic diet in children, mean (SD)**				
Baseline	2.2 (0.3)	2.3 (0.3)	NA	NA
No. of participants	371	373	NA	NA
4 mo	2.2 (0.3)	2.2 (0.2)	−0.02 (−0.06 to 0.01)	NA
No. of participants	290	288	NA	NA
12 mo	2.2 (0.3)	2.2 (0.2)	0.01 (−0.03 to 0.05)	NA
No. of participants	282	290	NA	NA
24 mo	2.2 (0.3)	2.3 (0.3)	−0.03 (−0.07 to 0.01)	NA
No. of participants	272	270	NA	NA
Pooled^c^	NA	NA	−0.02 (−0.05 to 0.01)	NA
**No. of caregiver toothbrushings per wk, mean (SD)**				
Baseline	11.7 (4.9)	11.9 (4.4)	NA	NA
No. of participants	377	377	NA	NA
4 mo	13.1 (3.8)	12.4 (4.2)	0.77 (0.21 to 1.34)	NA
No. of participants	290	288	NA	NA
12 mo	12.7 (4.2)	12.5 (4.4)	0.28 (−0.29 to 0.85)	NA
No. of participants	282	290	NA	NA
24 mo	12.7 (3.8)	12.3 (4.5)	0.36 (−0.22 to 0.94)	NA
No. of participants	272	270	NA	NA
Pooled^f^	NA	NA	0.48 (0.03 to 0.92)	NA
**Caregivers meeting guidelines for toothbrushing, No./total No. (%)**				
Baseline	227/377 (60.2)	228/377 (60.5)	NA	NA
4 mo	208/290 (71.7)	191/288 (66.3)	NA	1.50 (0.91 to 2.46)
12 mo	195/282 (69.2)	183/290 (63.1)	NA	1.51 (0.92 to 2.48)
24 mo	188/272 (69.1)	175/270 (64.8)	NA	1.34 (0.81 to 2.23)
Pooled^f^	NA	NA	NA	1.45 (1.04 to 2.03)

Abbreviations: CWT, child wellness text; NA, not applicable; OHT, oral health text; OR, odds ratio; SSB, sugar-sweetened beverage.

^a^ OHT effect is the mean difference between OHT and CWT, controlling for baseline value and child age.

^b^ OR for the outcome event, OHT compared with CWT, controlling for baseline value and child age.

^c^ Pooled effect from mixed-effects linear regression of data from 4, 12, and 24 months, controlling for baseline value and child age.

^d^ No pooled result given because of the significant interaction between intervention and time, indicating that the OHT effect changed over time.

^e^ Higher consumption was greater than 4.5 ounces compared with 4.5 ounces or less.

^f^ Pooled effect from mixed-effects linear regression of data from 4, 12, and 24 months, controlling for baseline value.

#### Preventive Dental Visits, Fluoride Use, Sugar-Sweetened Beverages, and Cariogenic Diet

Children of caregivers in the OHT group had significantly greater odds of having a preventive dental visit compared with children of caregivers in the CWT group, as indicated by pooled analyses over the 24-month follow-up period (OR, 1.51 [95% CI, 1.18-1.94]). Children of caregivers in the OHT group had significantly greater odds of using fluoride toothpaste compared with those in the CWT group, as indicated by pooled analyses over the 24-month follow-up (OR, 1.46 [95% CI, 1.06-2.01]). There were no significant differences between treatment groups on sugar-sweetened beverages or the consumption of cariogenic foods over time (Table 4).

#### Caregivers Brushing Their Own Teeth

Caregivers in the OHT group brushed their own teeth a significantly greater number of times per week (pooled mean difference, 0.48 [95% CI, 0.03-0.92]). Caregivers were more likely to meet toothbrushing guidelines (pooled OR, 1.45 [95% CI, 1.04-2.03]) compared with those in the CWT group (Table 4).

## Discussion

To our knowledge, this study is the first randomized clinical trial on text messaging to improve oral health in an at-risk population using a fully dose-matched comparison condition, an objective clinical outcome, and a 24-month follow-up. Although there was no treatment effect on new caries, OHT messages outperformed CWT messages on oral health behaviors that are well known to reduce caries, such as toothbrushing, engaging in preventive dental visits, use of fluoride toothpaste, and caregivers’ own brushing behavior. Previous pediatric oral health interventions have found changes in oral health knowledge but not in oral health behaviors or caries.^29,30^ While it is well documented that parent behaviors decrease children’s caries risk,^31^ it is possible that behavioral interventions with clinical caries outcomes may require longer than a 24-month follow-up since the effects of behavior change take time and have individual variability. Although over two-thirds of our sample was below the poverty line, engagement rates were high. Recruiting at-risk participants at medical clinics, coupled with high satisfaction, very low dropout rates, bilingual texts, and positive effects on oral health behaviors, suggests a high potential for dissemination.

Our study design was rigorous in that both groups were matched on program structure and features, leaving only the content to vary between groups. Moreover, few studies have shown a positive impact on toothbrushing among young children. A recent review found that only 19 of 42 studies were randomized clinical trials, and of those, only 7 showed improvements in toothbrushing compared with controls.^32^ Our study demonstrated significant, sustained improvements over 24 months in the quantity of toothbrushing, as well as fluoride toothpaste use, which are 2 complementary behaviors that reduce childhood caries.

Dental visit attendance by age 1 year has been shown to increase access to preventive services and to decrease future caries and dental costs.^33^ However, prior studies have shown that less than half (43%) of parents are aware of this recommendation, and of those who are aware, two-thirds (67%) disagree with it or state that a health care practitioner told them that a dental visit at age 1 year is unnecessary (22%).^34^ Despite these gaps, there is a paucity of interventions targeting parents to increase dental visits for young children.^28^ In the current study, children in the OHT group were over 50% more likely to have a preventive dental visit compared with those in the CWT group (pooled OR, 1.51 [95% CI, 1.18-1.94]).

### Strengths and Limitations

The strengths of this study include a fully dose-matched comparator, a bilingual program, longitudinal follow-up periods to 24 months, ongoing clinical examiner calibration, and mapping the intervention to an underlying theory. Our study has a high level of generalizability owing to low selection bias (77.8% of those eligible were randomized) and enrollment of a diverse population.

A study limitation is the lower-than-expected OHA attendance, largely due to COVID-19. In addition, toothbrushing was self-reported, but the timeline follow-back technique has been shown to be a valid method of assessment with limited recall bias.^17,18,35^ Results should also be interpreted in light of the multiple time-effect comparisons made without adjustment.

## Conclusions

In this randomized clinical trial, a parent-targeted OHT messaging program was not effective at reducing dental caries but improved preventive dental caries behaviors among a population of underserved children and their caregivers. This intervention, which is low burden and is easily integrated into people’s lives, has the potential to reduce caries-related oral health inequities.

## Notes

Supplement 1.
Trial Protocol

Supplement 2. **eMethods.** Examples of Text Messages by Module **eAppendix.** COVID-19 Analytic Considerations: Addendum to Study Protocol

Supplement 3.
Data Sharing Statement
