# Supplementary material for: Parent-Targeted Oral Health Text Messaging for Underserved Children Attending Pediatric Clinics: A Randomized Clinical Trial
Source: JAMA Netw Open. 2025 Jan 2;8(1):e2452780. doi: 10.1001/jamanetworkopen.2024.52780 (PMC11696445; doi:10.1001/jamanetworkopen.2024.52780)
Supplement: Supplement 1. — Trial Protocol [file jamanetwopen-e2452780-s001.pdf]

Randomized Controlled Trial of Interactive Parent-Targeted Text Messaging in Pediatric Clinics to reduce Caries among Urban Children

NIDCR Protocol Number: 17-083-E

NIDCR Grant Number: UH3DE025492-03

Principal Investigator: Belinda Borrelli, PhD (Lead)

Co-Principal Investigator: Michelle Henshaw, DDS, MPH

NIDCR Program Official: Darien Weatherspoon, DDS, MPH

NIDCR Medical Monitor: Kevin McBryde, MD

Draft or Version Number: 2.1

29 March 2019

STATEMENT OF COMPLIANCE

The study will be conducted in accordance with the International Conference on Harmonisation guidelines for Good Clinical Practice (ICH E6), the Code of Federal Regulations on the Protection of Human Subjects (45 CFR Part 46), and the NIDCR Clinical Terms of Award. All personnel involved in the conduct of this study have completed human subjects protection training.

SIGNATURE PAGE

The signature below constitutes the approval of this protocol and the attachments, and provides the necessary assurances that this trial will be conducted according to all stipulations of the protocol, including all statements regarding confidentiality, and according to local legal and regulatory requirements and applicable US federal regulations and ICH guidelines.

Principal Investigator or Clinical Site Investigator:

Signed: ______________________________ Date: ______________

Name: Belinda Borrelli, PhD

Title: Professor

Signed: ______________________________ Date: ______________

Name: Michelle Henshaw, DDS, MPH

Title: Professor

TABLE OF CONTENTS

PAGE
STATEMENT OF COMPLIANCE 1
SIGNATURE PAGE 2
TABLE OF CONTENTS 3
LIST OF ABBREVIATIONS 6
PROTOCOL SUMMARY 8
1 KEY ROLES AND CONTACT INFORMATION 12
2 INTRODUCTION: BACKGROUND INFORMATION AND SCIENTIFIC RATIONALE 14
2.1 Background Information 14
2.2 Rationale 14
2.3 Potential Risks and Benefits 15
2.3.1 Potential Risks 15
2.3.2 Potential Benefits 16
3 OBJECTIVES 17
3.1 Study Objectives 17
3.2 Study Outcome Measures 17
3.2.1 Primary 17
3.2.2 Secondary 18
4 STUDY DESIGN 21
5 STUDY ENROLLMENT AND WITHDRAWAL 22
5.1 Subject Inclusion Criteria 22
5.2 Subject Exclusion Criteria 23
5.3 Strategies for Recruitment and Retention 24
5.4 Treatment Assignment Procedures 27
5.4.1 Randomization Procedures (if applicable) 27
5.4.2 Masking Procedures (if applicable) 27
5.5 Subject Withdrawal 27
5.5.1 Reasons for Withdrawal 27
5.5.2 Handling of Subject Withdrawals or Subject Discontinuation of Study Intervention 28
5.6 Premature Termination or Suspension of Study 28
6 STUDY INTERVENTION 29
6.1 Study Behavioral or Social Intervention(s) Description 29
6.2 Administration of Intervention 29
6.3 Procedures for Training Interventionists and Monitoring Intervention Fidelity 29
6.4 Assessment of Subject Compliance with Study Intervention 30
7 STUDY SCHEDULE 31
7.1 Eligibility Determination 31
7.2 Enrollment/Baseline 31
7.3 Follow-up 32
7.4 Final Study Visit (24 months + 31 days) 32

7.5 Early Termination Visit....33
7.6 Unscheduled Visit....33
8 STUDY PROCEDURES /EVALUATIONS....34
8.1 Study Procedures/Evaluations....34
8.2 Laboratory Procedures/Evaluations....36
9 ASSESSMENT OF SAFETY....37
9.1 Specification of Safety Parameters....37
9.1.1 Unanticipated Problems....37
9.1.2 Adverse Events....37
9.1.3 Serious Adverse Events....37
9.2 Time Period and Frequency for Event Assessment and Follow-Up....38
9.3 Characteristics of an Adverse Event....38
9.3.1 Relationship to Study Intervention....38
9.3.2 Expectedness of SAEs....38
9.3.3 Severity of Event....38
9.4 Reporting Procedures....39
9.4.1 Unanticipated Problem Reporting to IRB and NIDCR....39
9.4.2 Serious Adverse Event Reporting to NIDCR....40
10 STUDY OVERSIGHT....41
11 CLINICAL SITE MONITORING....42
12 STATISTICAL CONSIDERATIONS....43
12.1 Study Hypotheses....43
12.2 Sample Size Considerations....43
12.3 Planned Interim Analyses (if applicable)....45
12.3.1 Safety Review....45
12.3.2 Efficacy Review....45
12.4 Final Analysis Plan....45
13 SOURCE DOCUMENTS AND ACCESS TO SOURCE DATA/DOCUMENTS....49
14 QUALITY CONTROL AND QUALITY ASSURANCE....50
15 ETHICS/PROTECTION OF HUMAN SUBJECTS....51
15.1 Ethical Standard....51
15.2 Institutional Review Board....51
15.3 Informed Consent Process....51
15.4 Exclusion of Women, Minorities, and Children (Special Populations)....53
15.5 Subject Confidentiality....53
15.6 Future Use of Stored Specimens and Other Identifiable Data....53
16 DATA HANDLING AND RECORD KEEPING....54
16.1 Data Management Responsibilities....54
16.2 Data Capture Methods....54
16.3 Types of Data....54
16.4 Schedule and Content of Reports....55
16.5 Study Records Retention....63
16.6 Protocol Deviations....63

17 PUBLICATION/DATA SHARING POLICY 64
18 LITERATURE REFERENCES 65

LIST OF ABBREVIATIONS

AE	Adverse Event/Adverse Experience
AH	Agile Health
BIA	Budget Impact Analysis
BMC	Boston Medical Center
BRFQ	Basic Research Factors Questionnaire
CC	Coordinating Center
CFR	Code of Federal Regulations
CHC	Community Health Center
CONSORT	Consolidated Standards of Reporting Trials
CRF	Case Report Form
CWT	Child Wellness Text Message Program
DCC	Data Coordinating Center
DHHS	Department of Health and Human Services
DMFS	Decayed, missing, and filled tooth surfaces
DSMB	Data and Safety Monitoring Board
eCRF	Electronic Case Report Form
EDC	Electronic Data Capture
EHR	Electronic Health Record
FDA	Food and Drug Administration
FFR	Federal Financial Report
FWA	Federalwide Assurance
GCP	Good Clinical Practice
GSDM	BU Goldman School of Dental Medicine
HIPAA	Health Insurance Portability and Accountability Act
IB	Investigator's Brochure
ICF	Informed Consent Form
ICH	International Conference on Harmonization
ICMJE	International Committee of Medical Journal Editors
IRB	Institutional Review Board
MOP	Manual of Procedures
N	Number (typically refers to subjects)

NIDCR	National Institute of Dental and Craniofacial Research, NIH, DHHS
NIH	National Institutes of Health
OCTOM	Office of Clinical Trials Operations and Management, NIDCR, NIH
OHRP	Office for Human Research Protections
OHT	Oral Health Text Message Program
PHI	Protected Health Information
PI	Principal Investigator
QA	Quality Assurance
QC	Quality Control
SAE	Serious Adverse Event/Serious Adverse Experience
SCT	Social Cognitive Theory
SOP	Standard Operating Procedure
UP	Unanticipated Problem
US	United States

PROTOCOL SUMMARY

Title: Randomized Controlled Trial of Interactive Parent-Targeted Text Messaging in Pediatric Clinics to reduce caries among Urban Children

Précis: The aim of this study is to test an interactive parent-targeted text message-based intervention to reduce caries among children < 7 years old, who are recruited from targeted pediatric clinics and receive pediatric care at those clinics. The design is predicated on the fact that sustainability of health behavior change is greatest if the intervention is integrated into existing channels (i.e., pediatric clinics) and woven into the fabric of people's lives (e.g., text messages). Parent-child dyads will be randomized to Oral Health Text Messages (OHT) or Child Wellness Text Messages (CWT). The two interventions are matched on text message dose, duration and features. Participants in both groups will receive text messages for four months (2 per day in month 1; one per day in months 2-4), as well as weekly and monthly assessments delivered through text messaging. All participants will have an additional one-month text message 'booster,' one year after baseline. Surveys to assess oral health knowledge, behaviors, mediators, and moderators will be given at baseline, mid treatment (2 months) end of treatment (4 months), one year, and 2 years after baseline. Caries will be assessed by clinical exam at baseline, and 12, and 24 months after baseline. Analyses will use longitudinal modeling strategies. We will collect quantitative and qualitative data for the budget impact analyses, assess mediators and moderators of the treatment effect, and assess changes in oral health behaviors for caregivers and their children.

Objectives: Primary: 1. To test the efficacy of the OHT intervention. Parents of children <7 years old who attend one of four urban pediatric clinics will be randomized to receive either OHT or CWT. Caries will be objectively measured through clinical exam. H_1: Those receiving OHT will have lower 24 month incidence of caries vs. those receiving CWT. 2. To assess the mechanisms through which intervention effects occur (mediators). H_2: OHT will differentially change Social Cognitive Theory constructs over time vs. CWT. We will test the mediational effect on caries. Mediators will be assessed via self-reported measures with good psychometric properties.
Secondary: 1. To perform budget impact analyses of pediatric clinic adoption of the OHT intervention to determine the financial consequences of adoption, diffusion, and sustainability.
2. Examine change in pediatric oral health behaviors (e.g. tooth brushing, diet, sugar sweetened beverages, fluoride toothpaste use, preventive dental visits, and 'lift the lip' oral assessment by parents).
3. To assess the effect of the intervention on the parent's oral health status, knowledge, attitudes, and behaviors (spill-over effect),
4. To identify subpopulations for whom the intervention has the greatest effect (moderators; e.g., race/ethnicity, insurance coverage,

	parent educational level, parent depressed mood, and child's prior caries experience, intervention dose/engagement).
**Population:**	Participants will be 850 parent-child dyads, consisting of a parent and his/her child under the age of 7 who is attending one of our four targeted pediatric clinics (see below). Participants in our pilot RCT (n=55) were 96.3% female; Mean parent age =31.1; 73.6% below poverty level; 14.6% < high school education, 67.3% black, 9.1% White, 14.5% Hispanic, 9.1% other; mean child age =2.8.
**Phase:**	Phase III
**Number of Sites:**	Four pediatric clinics in urban and low income areas of Boston will serve as recruitment sites. Three sites are community health clinics (CHCs, Dorchester House and Codman Square and South End Community Health Center) and one site is a pediatric clinic in an urban safety net hospital (Boston Medical Center).
**Description of Intervention:**	We will recruit caregivers who speak either English or Spanish and have a child under the age of 7 years who attends one of our pediatric clinic partners. Caregivers who screen eligible will be consented into the study and complete a baseline survey. The index child will receive a baseline oral clinical exam. After these activities are completed, the parent will be enrolled into the study and randomized to receive either the OHT or the CWT intervention. Follow-up surveys will be administered through electronic and/or mobile platforms (e.g email, smartphone, or telephone) at 2- month (mid-treatment), 4- month (end of treatment), 12 and 24- month post baseline. Follow-up oral clinical examinations will occur at 12 and 24- months post baseline. During the 4- month TM intervention, weekly and monthly assessments will also occur through the TM program. A TM intervention 'booster' period of one month will also occur 12- months post baseline, after the 12 month assessment. Program features (quizzes, customization, personalization, tailoring, interactivity, problem solving photos/video, goal setting, and feedback and rewards), program structure (core and choice topics, assessments), engagement strategies (quizzes, rewards) and language preferences (English or Spanish) will be similar between the CWT and OHT. Both study groups will also have three 'weekly challenges' over the 4- month intervention program, in which they can earn extra rewards and 'unlock' secret characters for performing the target behavior (OHT=brushing; CWT=reading).
**Study Duration:**	3 years
**Subject Participation Duration:**	2 years

**Estimated Time to Complete Enrollment:**	12 months

Schematic of Study Design:

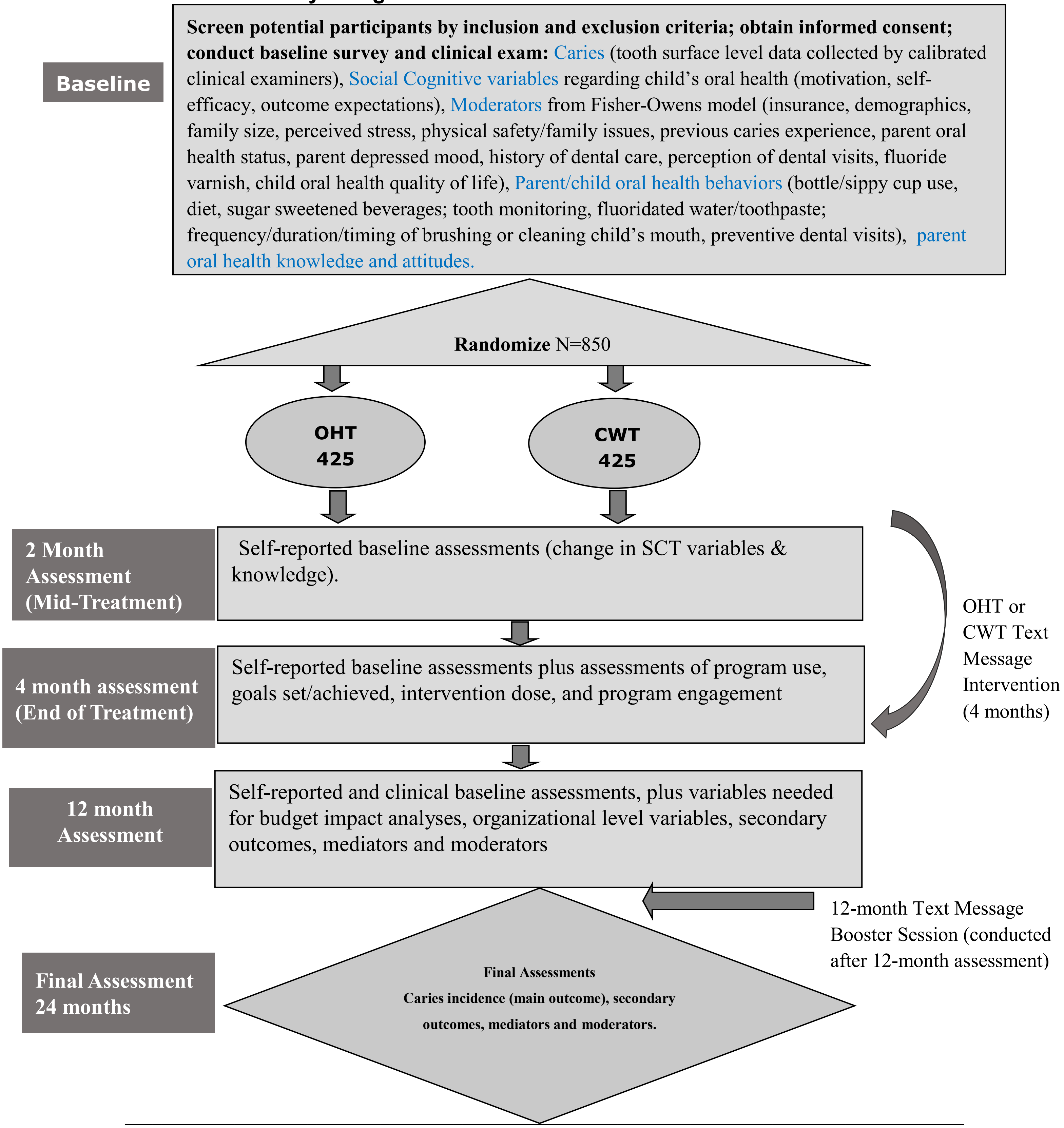

1 KEY ROLES AND CONTACT INFORMATION

Principal Investigator:

Belinda Borrelli, Ph.D., (Lead PI)
Professor, Boston University
Henry M. Goldman School of Dental Medicine
560 Harrison Avenue, 3rd floor
Boston, MA 02118
PH: 617-358-3358; FAX: 617-638-6381
belindab@bu.edu

Michelle Henshaw, DDS, MPH (Co-PI)
Boston University
Henry M. Goldman School of Dental Medicine
560 Harrison Avenue, 3rd floor
Boston, MA 02118
PH: 617-358-6111; FAX: 617-638-6381
mhenshaw@bu.edu

Medical Monitor:

Kevin D. McBryde, MD
Medical Officer
Division of Extramural Research
National Institute of Dental and Craniofacial Research
6701 Democracy Blvd., Rm. 638
Bethesda, MD 20892-4878
PH: 301-594-0170; FAX: 301-480-8319
mcbrydekd@mail.nih.gov

NIDCR Program Official:

Darien Weatherspoon, DDS, MPH
Director, Health Disparities Research Program
National Institute of Dental and Craniofacial Research
6701 Democracy Blvd., Room 640
MSC 4878
Bethesda, MD 20892-4878
Bethesda, MD 20817 (overnight mail or courier service)

PH: 301-594-5394; FAX: 301-480-8322
darien.weatherspoon@nih.gov

Clinical Site Investigators:

William Adams, M.D., Professor
Boston University School of Medicine, General Pediatrics
88 East Newton Street, Vose Bldg., 3rd Floor
Boston, MA 02118
PH: 617-414-7707; FAX: 617-414-3687
badams@bu.edu

Timothy Heeren, Ph.D., Professor
Boston University School of Public Health, Biostatistics
801 Massachusetts Avenue, Crosstown Center
Boston, MA 02118
PH: 617-638-5172; FAX: 617-638-6484
tch@bu.edu

Institutions:

Trustees of Boston University, BUMC
Office of Sponsored Programs
85 East Newton Street, M-921
Boston, MA 02118-2340
Diane Baldwin
Assistant Vice President, Sponsored Programs
PH: 617-638-4600; FAX: 617-638-4686
bumc-era@bu.edu

2 INTRODUCTION: BACKGROUND INFORMATION AND SCIENTIFIC RATIONALE

2.1 Background Information

The prevalence of caries in children is disproportionately high in racial and ethnic minorities (Dye et al., 2017). Pediatric clinics in urban and low income neighborhoods are an ideal venue to connect with parents who might not otherwise receive information on evidence-based oral health care for their child. Mobile phone ownership is near saturation (95%) (Pew Research Center, 2017) and >85% of adults text message (TM), with no disparities by income, education, or ethnicity (Pew Research Center, 2017; Duggan & Rainie, 2012; Bennett et al., 2012; Fox and Duggan, 2012; Zickuhr & Smith, 2012). The average number of daily texts is high (African-Americans 70.1; Latinos 48.9; Whites 31.2; Smith, 2011; Zickuhr & Smith, 2012). Using text messaging as an intervention platform and pediatric clinics as a channel for intervention could be an effective way to reach low income and ethnically diverse parents with an oral health behavior and caries prevention intervention. Our program is called 'iSMILE' (Interactive Short Messages to Initiate Lasting Health Education). Throughout the protocol iSmile is used interchangeably with "RCT of Interactive Parent-targeted text messaging in Pediatric Clinics to Reduce caries" when referring to this study. Our study includes a theory-based, fully powered dose-matched design with objectively measured outcomes and hypothesized mediators. The primary aim of the study is to test the efficacy of the Oral Health Text (OHT) message intervention by randomizing parents of children <7 years old attending pediatric clinic visits to receive either OHT or Child Wellness Texts (CWT). We will also examine a behavioral 'ripple effect' from the intervention (i.e., improvement in the parent's oral health behaviors), involve stakeholders on our advisory board, interview clinic decision makers, determine ways to integrate the intervention into work-flow and electronic health records (EHR), and perform budget impact analyses to aid in diffusion to other clinics. Thus, our multi-level project includes children, parents, providers, and clinic leadership.

2.2 Rationale

Rationale for platform. Text message interventions have been shown to be effective for changing health behaviors (Mason et al., 2015; Free et al., 2011; Saffari et al., 2014; Siopis et al., 2015; Finitsis et al., 2014) and are even more effective when integrated into clinic settings, such as pediatric clinics. Previous studies have shown that TMs have the following benefits: 1) access anytime/place, 2) ability to tailor content, timing, and intensity to users, 3) provision of real time coping strategies to users in everyday settings, 4) ability to support behavior change at key times, 5) few barriers to participation, 6) decreases the space/time gap between treatment and behavior, 7) interactive functionality in real time, 8) low participant burden, 9) reduces health care system cost (Chen et al., 2012; Guerriero et al., 2013), 10) ability to deliver intervention at key times (e.g., reminder to brush child's teeth), 11) instant support and feedback, and 12) potential for cost effective dissemination. Given that Blacks and Hispanics have the highest TM use, are likely to use texting to get health information, and also have children at highest risk for caries, this is an appropriate population in which to test a TM-based intervention intended to decrease caries incidence.
Text Messaging vs. Other technologies: 1) Mobile apps: Text messaging is superior in terms of popularity, simplicity, and world-wide scalability, because it is one of the most stable, transferable, cross-platform (phone, tablet, computer, etc.) methods available. Unlike apps, TMs do not require smartphones, and do not require users to download a program and accept 'push'

notifications. Only 53% of mobile phone owners and 29% of low income adults have smart phones (Fox & Duggan, 2012). Text messaging eliminates barrier points to entry by allowing anyone with a mobile phone to participate, regardless of make, model, or carrier. Users do not need to download anything to join. Also, text messaging is a conversation. A ‘push’ notification, such as with apps, is a one-way communication channel and users cannot respond directly. This creates a non-interactive experience, increasing the likelihood the user will turn off the push notifications or stop checking the app. Text messaging is highly interactive and inherently a two-way conversation. Users can text back and forth with our system, increasing engagement. 2) Web-based interventions: Short and low effort communications, such as text messaging, might be better for those who are less motivated to change. Web based interventions require more effort so less motivated parents are less likely to use them (Borland et al., 2013). Web-based studies on oral health promotion have had low usage rates (Hurling et al., 2013). TMs have wider reach than other electronic interventions (Abroms et al., 2012), have the advantage of instant transmission, and can prompt behavior at key times (e.g., nighttime brushing). Rationale for TM dose and duration. TM programs need to be of sufficient intensity and duration to affect behavior change, without being so burdensome that they cause drop out. We chose a 4 month intervention because most studies with interventions lasting >3 months had better outcomes (Siopis et al., 2015; Brendryen & Kraft et al., 2008; Celik et al., 2014). In addition, our pilot data indicate that the length of the program is satisfactory to our target population. Because caries prevention involves numerous target behaviors, it necessitates a longer and more intense intervention than health behaviors that are focused on changing one behavior (e.g., smoking cessation).

2.3 Potential Risks and Benefits

2.3.1 Potential Risks

This study poses minimal risk to the participant. Listed below are the risks:

- The loss of privacy and confidentiality.
- Some aspects of the assessments (e.g., answering questions about child care practices or the child sitting for the oral health assessment) may be uncomfortable or embarrassing.
- Untreated dental disease may be identified during the oral health assessment which may cause anxiety for the caregiver.
- There are two potential consequences of text messaging: accidents and thumb and joint pain. Texting while driving or walking could increase the risk of accidents. Frequent texting may increase the risk of thumb and joint pain. The number of texts involved in this study is not likely to result in thumb and joint pain.

“Agile Health” (AH) is the company that will be programming our TM curriculum. Their database will contain the TMs including information on what TMs each participant has received, which texts participants have responded to, their actual responses, and feedback about individual messages. This database is HIPAA secured.

Confidentiality will be maintained by numerically coding all data with a unique identifier. All electronic data will be stored in password protected, secured computer systems at the CC. Data will be collected through 1) secure electronic data capture (EDC) 2) paper and pencil questionnaires (only used in the event of EDC failure). Only the participant's study identification number will appear on any paper data or transcripts. Any paper data will be placed in a locked file cabinet. The master-code (or key), that links the subject name with subject's study identification number will be saved to the password protected electronic file, saved on the Dental IT approval secure S drive; this will be saved separately from the actual study data (e.g., screener and survey data). Data will also be collected through the AH text message platform, which is HIPAA secure. These data will not be de-identified, because identifying parameters are needed to initiate and customize the program. The identifying parameters include parent first and last name, child first and last name, parent gender, child gender, child date of birth, parent date of birth, zip code, mobile number, and preferred language to send/receive text (English or Spanish). Data collected through the TM program that are identifiable are caries history, toothbrushing practices, reading practices, text message use, text message responses, motivation and confidence to engage in health habits, dental visits, child safety, and other child health and wellness behaviors.

Participant information will be accessible only to research staff. Individually identifiable information will be destroyed seven years after the study has been concluded.

It is possible that some aspects of the assessments or TMs (e.g., answering questions about child care practices) may be uncomfortable. Participants will be informed that they can refuse to answer any questions or text messages that make them uncomfortable. Participants will be clearly informed of the topics to be addressed when receiving the TMs: e.g. brushing your child's teeth, visiting the dentist, etc. If a child becomes uncomfortable or overly anxious at the time of the screening, child dissent will be honored by the clinical examiners and the screening will be stopped.

All caregivers will receive a "report card" providing a summary of untreated dental decay found and a recommendation regarding the timing for seeking care. All caregivers are offered help in making a dental appointment and study staff will proactively follow up in cases with pain or infection to facilitate dental treatment.

To prevent TM related injury, participants will be told that they should not view, send, or receive texts while driving or walking. During the pilot phase, there were no adverse events reported.

2.3.2 Potential Benefits

Because both groups receive educational TMs, all participants may benefit (i.e., there is no 'assessment only' control group). Participants may have increased knowledge about child health, changed attitudes about child health, and/or perform healthy behaviors to facilitate child health. Another potential benefit is that the child receives a brief oral health exam with possible referral.

3 OBJECTIVES

3.1 Study Objectives

Primary Aims:

1. To test the efficacy of the OHT intervention in a theory-based, fully powered, dose-matched design with objective clinical measurement of caries. Parents of children <7 years old attending one of our partner pediatric clinics will be randomized to receive either OHT or CWT. H_1: Those receiving OHT will have lower 24- month incidence of caries vs. those receiving CWT, as assessed by clinical exam. Clinical exams will take place at baseline and 12-and 24- months later.

2. To assess the mechanisms through which intervention effects occur (mediators). H_2: OHT will differentially change Social Cognitive Theory constructs (motivation, self-efficacy, outcome expectations) over time vs. CWT. We will test the mediational effect on caries. Mechanisms will be assessed through text messaging assessments during the intervention, and through self-report surveys at baseline, and 2, 4, 12 and 24- months after baseline.

Secondary Aims:

1. To perform budget impact analyses of pediatric clinic adoption of the OHT intervention to determine the financial consequences of adoption, diffusion, and sustainability. The budget impact analyses will include interviews with key stakeholders.

2. Examine change in pediatric oral health behaviors (e.g. tooth brushing, diet, sugar sweetened beverages, fluoride toothpaste use, preventive dental visits, and ‘lift the lip’ oral assessment by parents). These variables will be assessed at baseline, 4, 12, and 24 months after baseline.

3. To assess the effect of the intervention on the parent’s oral health status, knowledge, attitudes, and behaviors (spill-over effect). These variables will be assessed at baseline, 4, 12, and 24 months after baseline.

4. To identify subpopulations for whom the intervention has the greatest effect (moderators; e.g., race/ethnicity, insurance coverage, parent educational level, parent depressed mood, child’s prior caries experience and intervention dose/engagement). All moderators will be assessed at baseline, except for intervention dose/engagement, which will be assessed during the course of text message delivery.

3.2 Study Outcome Measures

3.2.1 *Primary*

Caries Incidence: Tooth surface level data in children, collected by calibrated clinical examiners (blind to treatment condition), utilizing the OHDC Modified ICDAS methodology, will allow calculation of caries incidence rates (development of a new cavitated lesion or filled surface in a previously sound tooth surface or tooth surface without a cavitated lesion). Data will be collected by clinical examiners at baseline, 12, and 24- months.

Mediators:

Self-efficacy to perform oral health behaviors (12 items). The overall self-efficacy score represents how sure participants are that they can engage in recommended behavior to take care of their children's teeth. The overall self-efficacy score is the mean response. Items are scored on a 1-7 scale, 1 = not sure at all to 7 = extremely sure. This measure will be assessed at all measurement points (baseline and 2, 4, 12, 24 months) (Cronbach's alpha = .92). A one-item measure of this construct will be assessed during the TM program.

Motivation to perform oral health behaviors (12 items). The overall motivation score represents how much participants want to engage in recommended behavior to take care of their children's teeth. The overall motivation score is the mean response. Items are scored on a 1-7 scale, 1 = do not want to at all to 7 = very much want to. This measure will be assessed at all measurement points (Cronbach's alpha = .88). A one-item measure of this construct will be assessed during the TM program.

Outcome expectations regarding oral health behaviors. This measure assesses the degree to which participants perceive that performing the oral health behaviors (e.g., brushing) will lead to the desired outcome (e.g., reduced dental caries) in their children (Stewart et al., 1997). This measure will be assessed at all measurement points. A one-item measure of this construct will be assessed during the TM program.

Number of goals set and completed will be assessed during the TM program. OHT will set goals to brush (or supervise brushing) their children's teeth and CWT will set goals to read to their children. Goals set and completed are automatically tracked in the AH TM platform system. We will also assess whether or not participants 'opt in' to participating in 'challenge weeks.' For OHT, this will entail setting a goal of brushing every day, twice per day. For CWT, this will entail reading to their child every day. Both groups will be assessed each day for their adherence.

3.2.2 Secondary

Budget impact analysis: Measures and analyses will follow best practice guidelines (Mauskopf et al., 2007). Dental public and private insurance claims will be collected from each participating site. Labor Costs: Labor time will be assessed for each dissemination scenario at each site and average salary for each personnel will be used to determine labor cost. We will measure costs of interface development, implementation, and technical support.

Pediatric oral health behaviors:

Brushing. We will assess frequency, duration, timing, and supervision of brushing using items from the BRFQ (Albino et al., 2017). This measure will be assessed at all measurement points, and during the TM program.

Bottle/sippy cup/cup use. This measure will be assessed at all measurement points, and during the TM program. We will use items from the BRFQ (Albino et al., 2017).

Diet (Food and Beverage Consumption Scale; Palmer et al., 2010; 31 food/beverage items). Participants are asked to assess their child's food intake by selecting the frequency in which each food item is consumed over the past month on a five-point Likert scale, which is scored from 0-4 and measured by the following choices: Never/Rarely; 1-2 times/week; 3-6 times/week; 1 time/day; 2 or more times/day. The food items presented are divided into 6 food categories: cereal; fruits and vegetables; grains, breads, and pastas; dairy; snacks and sweets; and drinks. Each food category contains approximately 2 or more foods or beverages. The estimated cariogenicity score is composed of 5 cariogenic categories, calculated for each child by obtaining a weighted sum of the number of food items for each food category and dividing by the total food and beverage items. This measure will be assessed at baseline, 4, 12, and 24 months after baseline.

Sugar sweetened beverage consumption (15 items). We will use the BEVQ-15 to assess sugar-sweetened beverages and total beverage energy intake (Hedrick et al., 2012). These measure will be assessed at baseline, 4, 12, and 24 months after baseline.

Tooth monitoring. We will assess the frequency of parental checking of child's teeth for early signs of cavities. This will be assessed at all measurement points. We will use items from the BRFQ (Albino et al., 2017). This measure will be assessed at baseline, 4, 12, and 24 months after baseline.

Fluoridated water/toothpaste. This measure will be assessed at baseline, 4, 12, and 24 months after baseline. We will use items from the BRFQ (Albino et al., 2017).

Preventive dental visits. This measure will be assessed at all measurement points, and monthly during the TM program. We will use items from the BRFQ (Albino et al., 2017).

Parental knowledge, attitudes and behavior regarding oral health:

Parent oral health knowledge: 21 items. This measure assesses parental knowledge related to oral health hygiene routines and feeding practices. The overall knowledge score represents the percentage of oral health knowledge items answered correctly. We will use items from the BRFQ (Albino et al., 2017).This measure will be assessed at baseline, 4, 12, and 24 months after baseline.

Parent's attitude toward their own and their child's oral health. We will use items from the BRFQ (Albino et al., 2017). This measure will be assessed at baseline, 4, 12, and 24 months after baseline.

Parent oral health behaviors. 12 items related to parental oral health behaviors, such as oral hygiene routines.

Moderators from Fisher-Owens model:

Demographics: Insurance coverage, income, education, ethnicity, and family size, This measure will be assessed at baseline. We will assess changes in mutable variables such as income and insurance at other measurement points. We will use items from the BRFQ (Albino et al., 2017).

Perceived stress (Herrero & Meneses, 2006): We will use the 4-item version of the perceived stress scale (PSS). The PSS assesses the degree to which subjects perceive their environment and their experiences as stressful. The scale correlates well with scores on life-event measures, and has demonstrated adequate reliability (Cohen et al., 1983). This measure will be assessed at baseline, 4, 12, and 24 months after baseline.

Physical safety/family issues (Romano et al., 1991): We will use the hassles scale which has been developed and validated on low income and ethnically diverse samples. The scale assesses neighborhood safety and family issues. This measure will be assessed at baseline, 4, 12, and 24 months after baseline.

Previous caries experience: This measure will be assessed at baseline, 12 and 24-months via clinical exam utilizing a modified ICDAS

Medical history: This will be assessed at baseline, 12 and 24 months by the clinical examiner.

Parent depressed mood: We will use the short-form of the Center for Epidemiologic Studies Depression Scale (CESD-R- 10; Kohut et al., 1993). The correlation between the long and short-forms is r=.91. Cronbach's alpha = .86. This measure will be assessed at baseline, 4, 12, and 24 months after baseline.

History of dental care (parent and child). This measure will be assessed at baseline, 12 and 24- months. We will use items from the BRFQ (Albino et al., 2017).

Perception of dental visits: We will assess parental attitude toward taking their child to the dentist. This measure will be assessed at all measurement points. We will use items from the BRFQ (Albino et al., 2017).

Fluoride varnish. This measure will be assessed at baseline, 12 and 24- months. We will use items from the BRFQ (Albino et al., 2017).

Child oral health quality of life. We will use the Child Oral Health Impact Profile-Reduced scale (SF-19), which shows good reliability and validity to measure oral health related quality of life (Broder et al., 2012). This measure will be assessed at baseline, 4, 12, and 24 months after baseline.

Intervention Dose and Engagement: number of texts sent; percent of texts responded to, number of unprompted responses, use of program features, topics chosen, and goals set/completed.

Program Satisfaction. We adapted the Mobile App Rating Scale (MARS; Stoyanov et al., 2015) to measure satisfaction with the text messaging program (e.g., would you recommend this program; '1'= not at all to '5' definitely). Our other measures of program satisfaction that we adapted from the MARS are: showing the TMs to others (yes/no); perceived helpfulness of TMs to family/friends; how much longer they want to receive the program; and satisfaction with each of the proposed program components (e.g., setting goals, choosing topics) and structure (e.g, time of day TMs received, frequency). The MARS also measures perceived impact of the intervention, and we adapted it for oral health. We measure the perceived impact of the program on their own attitudes towards their child's oral health and oral health practices.

Perceived impact on their own attitudes towards their child's oral health is measured with six items on a scale of 1 (strongly disagree) to 5 (strongly agree; e.g., iSMILE has improved my attitude toward my child's oral health) Perceived impact on oral health practices are measured with six items on a scale of 1 'not at all' to 10 'very much.' (e.g., 'How much do you think that iSMILE had a positive impact on decreasing the amount of sugar sweetened beverages your child drinks."). This measure will be assessed immediately after the conclusion of the fourth month text message program (at the 4-month follow-up).

4 STUDY DESIGN

This is a 2-arm, randomized controlled Phase III, single-center longitudinal trial. 850 parents or caregivers of children (< 7 years old) and their children with pediatric appointments at four targeted urban clinics will be recruited, consented, and subsequently randomized to OHT or CWT. We hypothesize that those randomized to the OHT group will have reduced incidence of caries as assessed by objective clinical exam 24- months after a baseline exam. Randomization will be a permuted block design, stratified by study site (Codman Square, Dorchester House, BMC, South End Community Health Center), and history of caries (no history of caries, any caries). Because of the sparsity of Spanish speaking participants, with Spanish speakers primarily concentrated at one site, we will not stratify by site language (English, Spanish) but only on caries history to yield 8 strata. CWT and OHT intervention arms will be matched on TM dose, duration and frequency, and employ identical features and engagement strategies for strong internal validity. External validity will be supported by the fact that participants will be proactively recruited from an existing channel (pediatric clinic appointments) and use of the text message program in their everyday lives. Recruitment sites are located in urban, low-income areas, and the population is ethnically diverse and on public assistance.

The approximate time to complete study enrollment is 1- year and the expected duration of subject participation is 2- years. A clinical exam to assess caries will take place at baseline and 12 and 24- months later. Clinical examiners are study staff and will be calibrated and blinded. Self-report surveys will take place at these time points, plus 2 and 4- months after baseline. These data will be captured through electronic data entry, in person, over the phone, or through an on-line link. Weekly and monthly assessments will also occur during the 4- month TM program (from baseline through 4-months post-baseline). The coordinating center (CC) will be University of California at San Francisco.

CWT or OHT TMs in either English or Spanish will be delivered in sets of two each day in Month 1, and one set per day in Months 2-4, exclusive of brief assessments by text. At month 12, participants will receive one month of 'booster' TMs (twice per day, exclusive of brief assessments by text). Features include: quizzes, customization, personalization, tailoring, interactivity, problem solving photos/video, goal setting, and feedback and rewards using a gamification approach. There will also be three 'weekly challenges' over the 4- month program in which both groups can 'opt-in' and earn weekly rewards and 'unlock' secret characters for performing the core targeted behavior (brushing and reading for OHT and CWT respectively). Target behaviors (Reading for CWT and Brushing for OHT) will be assessed daily via TMs during challenge weeks. Otherwise, both groups will be assessed weekly via TM on core topics (brushing for OHT; reading for CWT), motivation and confidence (to brush or to read). Monthly assessments include dental visits (OHT) or safety (CWT). Both groups will have the ability to choose 'choice topics' each week and will receive the same engagement strategies (quizzes, rewards, birthday texts).

CWT include the Core Topics Reading and Child Safety, and the Choice Topics Physical Activity, Healthy Development, Secondhand Smoke, Safety Hazards, Sleep and Behavior, and Stress tips for parents. CWT participants earn weekly badges for reading to their child. Participants who are not reading to their child at recommended levels will received a graduated reward scheme, in which they will receive rewards for successive approximations of the recommended behavior. The goal is to engage with reading to their child every day. If they earn four different weekly badges (each badge specifying the reading goal achieved), they will earn a different 'Book Buddy' badge each month. They can also earn two 'mystery book buddies' for the completion of challenge weeks (Book buddies are anthropomorphized books).

OHT include the Core topics Brushing and Visiting the Dentist, and the Choice topics Bedtime routine, Bottle/Sippy cup use, Sugar Sweetened Beverages, Healthy Eating, Getting Fluoride and Fun Facts. OHT participants earn weekly badges for brushing (or supervising brushing) their child's teeth. Participants who are not brushing (or supervise brushing) their child's teeth at recommended levels will received a graduated reward scheme, in which they will receive rewards for successive approximations of the recommended behavior. The goal is to brush their child's teeth (or supervise brushing) 6-7 days per week, twice per day. If they earn four different weekly badges (each badge specifying the brushing goal achieved), they will earn a different 'SuperTooth Hero' badge each month and collect an entire family of SuperTooth heroes (Charlie Chew, Molly Molar, Faye Fluoride and Captain Chomp). They can also earn two 'Secret Super Tooth Heroes' for the completion of challenge weeks. SuperTooth Heroes are anthropomorphized teeth wearing capes and holding toothbrushes.

<u>Substudies</u>

Because our comparison group (CWT) is an intervention, we would like to assess within- and between subject changes over time to gather pilot data for a future trial. The aim of this substudy would be to examine whether CWT parents increase their behaviors in two areas that reflect the core topics of the CWT intervention: reading to their child and child safety. Core topic text messages are given once per day during month one (choice topics are also given once per day in month one) and 4 days per week during months 2-4 (choice topics are given 3 days per week during months 2-4). Choice topics for CWT are: Physical Activity, Healthy Development, Secondhand Smoke, Safety Hazards, Sleep and Behavior, and Stress tips for parents. This substudy will have no impact on the main study. We will analyze data from all participants in CWT and examine within subject changes over time, as well as whether or not they differentially changed in key variables (reading, safety) vs. OHT. a separate analysis plan will be developed in the future.

5 STUDY ENROLLMENT AND WITHDRAWAL

5.1 Subject Inclusion Criteria

In order to be eligible to participate in this study, the caregiver and child must meet all of the following criteria, as applicable:

1. Provide informed consent for themselves and their child.
2. Willing to comply with all study procedures and be available for the duration of the study
3. Caregiver may be male or female, must be aged 18 or older (no upper age limit).

4. Caregiver must be a parent or legal guardian of a child less than 7 years old
5. Enrolled child must have their first tooth showing.
6. The child must receive medical care at one of the participating pediatric clinics (BMC, Dorchester House, Codman Square, South End Community Health Center).
7. Speak, understand, and read either English or Spanish fluently.
8. Have a mobile phone.
9. Have used any type of text messaging at least one time in the past month.
10. Have a mobile phone plan that includes unlimited text messaging.
11. Score above the literacy cut-off, i.e. score less than or equal to 3 on the Single Item Literacy Screener (Morris et al., 2006)
12. Ability to receive a test TM from the Agile Health system and 'opting-in' to program

5.2 Subject Exclusion Criteria

If the caregiver or child meets any of the following criteria, the dyad will be excluded from participation in this study:

1. Participated in our usability trial or RCT pilot trial
2. Substance abuse problems, as identified with the AUDIT-C screening tool and with questions about drug misuse.
3. Diagnosis of serious mental illness (Bipolar Disorder, Mania, Manic-depressive disorder, Schizophrenia, Psychosis), self-reported.
4. Children with severe congenital tooth malformations: at screening the caregiver will be asked if their child has known systemic diseases associated with abnormal tooth development or abnormal oral health status such as cleft lip or palate, amelogenesis imperfecta, or dentinogenesis imperfecta.
5. Currently enrolled in another mobile phone study.
6. Plan on moving out of the area during the next 2 years.

7. Plan on leaving the country for longer than two months during the 2- year study.

8. Enrolled in an oral health study or child health and wellness study.

9. Exclusion at the discretion of the Principal Investigator

10. Children who cannot complete the baseline oral health exam.

5.3 Strategies for Recruitment and Retention

The study target population includes parents or legal guardians (>18 years old) of children (< 7 years old) and their child with a pediatric appointment at one of the four participating clinical sites.

The primary recruitment method for the study will be RAs, or other trained study personnel, identifying and recruiting participants in participating clinic waiting rooms (and other clinic spaces deemed appropriate by clinic partners).

Additional strategies to identify potentially eligible participants may vary by site but include:
(1) The site specific study champions and clinic staff and providers will identify potentially eligible participants, inform them about the study and direct them to a RA. If the RA isn't present, the clinic staff will give the participant a study pamphlet and/or permission to contact form for the iSmile study. The permission to contact form may be printed with pre-populated the patient's information (contact details), or given to the patient as a blank form.
(2) iSMILE study staff will present the study at each clinical site at all-staff meetings to facilitate clinical staff and provider referral of potentially eligible participants to the iSMILE study;
(3) Bilingual study advertising, with RA contact information, will be on display in study clinics;
(4) Bilingual study advertising inviting interested clinic patients to attend study information session phone calls to learn more about the study;
(5) "Warm-hand-off" phone calls will be conducted by the clinic-employed study champion to potentially eligible participants prior to their scheduled clinic appointment to confirm their upcoming appointment and determine interest in the study (no cold calls will be made by our research staff). If the person is interested, the person will be referred to our study staff;
(6) Partnering with other studies happening at participating clinics to facilitate referrals to iSmile;
(7) Sending an informational letter from the Pediatric Medical Director to potential participants.
(8) Distributing recruitment materials (flyers, brochures and permission to contact forms) at Head Start locations because many parents who visit Head Start also bring their children to receive care at one of our recruitment sites.

Conducting "warm-hand-off" phone calls prior to a potentially eligible participants scheduled clinic visit will allow individuals the ability to plan for the extra time needed at their appointment to enroll and complete baseline study procedures. Research staff will identify subjects eligible for "warm-hand-off" phone calls from participating clinics' appointment schedule (part of the electronic health record (EHR). Research staff will have 'read-only' access to the EHR and they will not have access to the medical portion of the record. The parts of the EHR that we will have access to contains information on upcoming clinic appointments (date/time), name of parent and child, contact information, and age of child. The "warm-hand-off" phone call between clinic personnel and RAs will involve a phone call from clinic personnel to a potentially eligible subject

to confirm their upcoming appointment and then determine their interest in learning more about the iSmile study. If the potentially eligible subject wants to learn more about the iSmile study over the phone, the clinical staff will hand the phone off to the iSmile RA.

The iSmile study team will partner with other studies happening at participating clinics to facilitate referrals to iSmile. The iSmile team has learned of the other co-occurring studies happening in each of the participating clinics that overlap with the target iSmile recruitment population. From these co-occurring studies, iSmile has worked with each applicable study contact to determine if cross recruitment is feasible. For those studies for which cross recruitment is feasible, co-occurring study staff members will screen their potential participants per their IRB approved protocol. For potential participants who screen ineligible for other co-occurring studies, the co-occurring study staff conducting screening will inform the individual that they may be eligible for the iSmile study. If an iSmile study staff member is available, the potential participant will be directly introduced to the iSmile study staff member to hear more about the study and potentially complete the IRB approved screening and enrollment process. If an iSmile study staff member is not available to tell the potential participant more about the study, the co-occurring study staff member will inform the potential participant that they can complete a paper permission to contact form and a member of the iSmile study staff will contact them shortly to tell them more about the study. Each participating clinical site has designated its own secure location for completed permission to contact forms to be stored on site until an iSmile study staff member is able to collect them. After collection, iSmile study staff will attempt to contact all potential participants to tell them more about the study. After successfully, or unsuccessfully, attempting to contact the potential participant, all completed permission to contact forms will be store in a locked filing cabinet in the Research Project Manager's office.

The informational letter being sent from each pediatric clinic, from the Pediatric Medical Director, will inform parents of children under age 7 that the health center is partnering with Boston University to develop a text message program. Study staff contact information is also included in the letter, should the parent be interested in assessing eligibility or asking questions about the study.

If participants do not have time to be screened at the clinic, we will collect their contact information and the participant will be screened over the phone. Participants who are called will be guided through the consent process over the phone and we will obtain verbal consent over the phone (seeking a waiver of documentation of consent) until they can provide signed consent at the time of the clinical exam. This is so participants can spend less time doing study procedures at the clinic. Clinic staff will also refer potentially interested participants to RAs.

Study staff will perform screening and informed consent (in a private space or on the phone, prior to their appointment). Participants will be told that, in order to be eligible for the study, they must be willing to receive TMs about their child's health, have a phone that is able to send/receive text messages from our system, give permission for the index child to have dental exam, opt-in to the program, and receive one of two different TM programs. If there is more than one child < 7 years old in the same family, we will systematically choose the youngest as the index child for the study. If there is more than one parent present, we will choose the one that spends the most time with the child. Those who are eligible to participate will be asked to sign an electronic informed consent form, complete contact information and child demographic information with study staff who will verbally collect information via direct entry, and subsequently complete a self-report baseline assessment via electronic data capture (with

paper copies as back-up) and the baseline oral assessment. Study staff will take all possible steps to ensure that the participant is clear that the baseline questionnaire will ask questions regarding themselves and the child in the study (youngest child under the age of 7 years old with at least one tooth showing i.e., child for whom the informed consent had been collected). Once the baseline questionnaire and oral assessment are completed, REDCAP will automatically randomize participants into OHT or CWT, and this code as well as the TM personalization options will be transferred to the AH system so the first TM (OHT or CWT) will occur within 24 hours after randomization. The automization of randomization will ensure that the RAs are blind to treatment condition.

The enrolled parent/caregiver will receive $60 for assessment contacts that include a self-report instrument and an oral health assessment conducted by the study clinical examiner (baseline and 12 and 24- months), $40 for assessment contacts which only include the self-report instrument (2 and 4- months after baseline), an additional $40.00 if the 12 and 24 month plus either the 2 or 4 month assessments are completed, or $70.00 if all 4 assessments are completed. Thus, the total possible compensation for each parent who is a participant is $330.00. If at any point during the study following baseline it is determined that a participant will not be able to complete the 12 or 24 month oral health exam because they moved out of the area, we would still like to compensate the participant for completing the 12 or 24 month follow up surveys without the oral health exam. The participant would then be compensated with a $40 gift card instead of $60, which is comparable to compensation at 2 and 4 months when participants are expected to only complete surveys. In addition, individuals who move out of the area and their situation is deemed by study research staff to be impossible to complete the oral health assessment are still eligible for bonus payment.

During the text message program, participants will be given brief weekly assessments. Each time they provide an answer to these questions, their name will be entered into a monthly raffle for $100.00. Every time that they answer one of these questions, they will have another entry into the raffle, increasing their chances of winning each month, for a maximum of four chances. Therefore, if they answer each of the four weekly questions per month, they will have four entries into the raffle that month. After the name is drawn for that month, a new month will begin and all previous entries will be cleared. Because they will receive text messages over the course of four months, they will have four chances to win.

Assessments will be completed online, on participant’s smartphone, on the phone, or in person.

To prevent dropout during the 4- month intervention period, we will personalize TMs, avoid TM redundancy, vary TM content and frequency, use participant’s name and use fun TM quizzes, with the answers provided the following day. After the intervention period, we will use various cohort maintenance procedures as outlined in Albino et al (2017) to enhance retention (e.g., send birthday cards, contact six times/year to find out address changes) and also track participants through their contact with the pediatric clinics (i.e. identification of upcoming child wellness appointments through the clinic’s electronic scheduling system. Guidance from the AB will also assist in the development of retention strategies.

In addition to direct compensation for their participation in the study, participants will also have be compensated for their transportation to and from participating clinical sites. As the Baseline

enrollment process is long, not all families who begin the enrollment process are able to complete it in one day. To help facilitate enrollment, the study will compensate participants in the form of a $10 gift card for their transportation to and from participating clinical sites for participants who need to return to complete their enrollment process in a second visit. In addition, the study will provide the same $10 gift card compensation t for participant transportation to and from participating clinical sites for enrollees to complete the 12 and 24 month oral health assessments at the clinic site.

5.4 Treatment Assignment Procedures

5.4.1. Randomization Procedures

In order to randomly assign participants to treatment groups at baseline, we will be using a permuted randomized block design (random sized blocks of 2 or 4 participants), stratified by study site (Codman Square, Dorchester, BMC, South End Community Health Center), and history of caries (no history of caries, any caries). Because of the sparsity of Spanish speaking participants, with Spanish speakers primarily concentrated at one site, we will not stratify by site language (English, Spanish) but only on caries history to yield 8 strata. The randomization list will be created using REDCap. Participants will be randomized by the web-based REDCap randomization module. Once the baseline questionnaire and oral assessment are completed, REDCap will automatically randomize participants into OHT or CWT, and this code as well as the TM personalization options from the baseline questionnaire will be transferred via secure API to the AH system so the first TM (OHT or. CWT) will occur within 24 hours after randomization. The automization of randomization will ensure that the RAs who have contact with participants are blind to treatment condition. The clinical examiners who will be collecting the primary outcome data will also be blinded. A detailed plan for maintenance of randomization codes, including the timing and procedures for planned and unplanned breaking of randomization codes is described in the MOP.

5.4.2 Masking Procedures

All study staff collecting data, including the clinical examiners and RAs, will be blind to treatment condition. The Project Director and PIs will not be blind to treatment condition, because they will need to monitor inbound text messages for any responses that the automated system does not recognize. Unmasking procedures are detailed in the MOP.

5.5 Subject Withdrawal

Subjects are free to withdraw from the study at any time. During the TM program, the participant can text ‘stop’ at any time and the messages will cease. After the TM program, participants can e-mail iSMILE, or call or text iSMILE, to state that they want to withdraw from the program. If a study subject requests to be withdrawn from the study, the research team will attempt to ascertain the reason for withdrawal. The Principal Investigators will also have the option to withdraw a participant if continued participation is not in the best interest of the participant.

5.5.1 Reasons for Withdrawal

We anticipate that reasons for a subject’s withdrawal may include one or more of the following:

- Does not have time to participate.
- Does not want to receive TMs.
- Does not feel the need for health information.
- His/her child is not in their custody at the present time.
- They are going out of the country permanently or for an extended period of time.

A study subject may be withdrawn from study participation by the investigative team if:

- Any clinical adverse event (AE), laboratory abnormality, or other medical condition or situation occurs such that continued participation in the study would not be in the best interest of the subject.
- The subject meets an exclusion criterion (either newly developed or not previously recognized) that precludes further study participation.

5.5.2 Handling of Subject Withdrawals or Subject Discontinuation of Study Intervention

We will attempt to obtain a final child oral health assessment and study questionnaires from all subject caregivers, including those who withdraw from the study for any reason. We will also attempt to perform an oral assessment on the child at the time of withdrawal if a subject withdraws early (before 24 months of participation). We will make a minimum of 5 attempts to contact the caregiver in cases of withdrawal and those who relocate. At the time of withdrawal, study staff will review procedures for contacting the study team in case an AE or UP occurs.

Once a participant has been randomized, and begins the program, replacement of that participant is not allowed if the participant withdraws prematurely. If the participant becomes ineligible (e.g., child no longer lives with them), then we will replace the subject but only if the study is within the specified recruitment period.

5.6 Premature Termination or Suspension of Study

This study may be suspended or prematurely terminated if there is sufficient reasonable cause. Written notification, documenting the reason for study suspension or termination, will be provided by the suspending or terminating party to investigator and NIDCR. If the study is prematurely terminated or suspended, the principal investigator will promptly inform the IRB and will provide the reason(s) for the termination or suspension. Circumstances that may warrant termination include, but are not limited to:

- Determination of unexpected, significant, or unacceptable risk to subjects.
- Insufficient adherence to protocol requirements.
- Data that are not sufficiently complete and/or evaluable.

- Determination of futility.

6 STUDY INTERVENTION

6.1 Study Behavioral or Social Intervention(s) Description

The intervention is an interactive, parent-targeted TM program to reduce caries among low income families visiting urban pediatric clinics. Caregivers and their children under the age of 7 attending pediatric visits at one of our clinic partners will be randomized to receive one of two dose-equivalent TM interventions: Oral Health Texts (OHT) or Child Wellness Texts (CWT). The intervention includes a theory based, fully powered dose-matched design with an objectively measured clinical outcome. The CWT intervention is matched to OHT intervention on: 1) number of core modules and ability to choose from a menu of options for additional topics, 2) number of TMs and program duration 3) engagement strategies and 4) program features. For the OHT group, the two core topics are Brushing and Visiting the Dentist. In addition to the required OHT Core topics, caregivers can choose from the following menu of topics: Bedtime Routine, Bottle/Sippy Cup Use, Sugar Sweetened Beverages, Healthy Eating, Getting Fluoride (tap water and toothpaste) and Fun Facts. For the CWT group, the two core topics are Reading and Child Safety. In addition to these required Core topics, caregivers can choose from the following menu of topics: Physical Activity, Healthy Development, Secondhand Smoke, Safety Hazards, Sleep and Behavior and Stress Tips for You.

6.2 Administration of Intervention

The TM intervention is automated and interactive. The program will begin sending TMs within 24- hours of randomization. Both OHT and CWT will be 4-months in duration, with a 1-month booster session that will occur 12 months after baseline (8 months after the end of the 4 month program). TMs will be delivered approximately twice each day in Month 1, and once per day in Months 2-4 (exclusive of brief assessment questions which occur several times per week). During the booster session (at 12 months after baseline), TMs will be delivered approximately twice per day, exclusive of brief assessment questions. Brief assessment questions will occur several times per week. The number of texts, dose, and duration will be equivalent between the two groups. Participants in both groups will choose ‘choice topics’ each week, and will be assessed weekly via TM on core topics (brushing for OHT; reading for CWT) and mediators (motivation, self-efficacy and outcome expectations). Monthly assessments include dental visits (OHT) or safety (CWT)

6.3 Procedures for Training Interventionists and Monitoring Intervention Fidelity

The intervention will not utilize human providers. TM interventions are delivered exactly as designed, resulting in a 100% reliable intervention, providing confidence in the obtained results. However, because the project director will be able to respond to TMs, we will review a proportion of the responses to ensure that there is no treatment contamination. Because our

outcome data is collected by a clinical examiner, this data collection will be digitally recorded by an RA to ensure that no oral health advice or education is given during that time.

6.4 Assessment of Subject Compliance with Study Intervention

The AH Platform keeps track of all TMs sent and received, including both unprompted responses and expected responses from participants. This includes responses to assessment questions, responses to weekly topic choices, and responses to the interactive intervention features of the TM programs. The study team will receive regular reports from AH as outlined in the MOP. The study team will review the reports and monitor compliance based on overall response rates and response rates to assessment questions.

7. STUDY SCHEDULE

The schedule of general study activities for individuals in both the intervention (OHT) and the dose matched control arm (CWT) is outlined in the following table. Once a subject has been determined eligible and has consented to study activities, a baseline survey and the clinical oral health assessment will occur. After baseline activities are completed, the intervention and control activities will be delivered via text messaging for 4- months. Study questionnaires will be administered at 2 months (mid treatment) and again at the end of the intervention (4 months). Study activities are described in greater detail below.

Experimental and Control	Baseline	2 M (-14 days or +31 days)	4 M (-14 days or +31 days)	12 M (-14 days or + 60 days)	24 M (-14 days or + 60 days)
Eligibility Screening (pre-baseline)	X				
Questionnaires	X	X	X	X	X
Oral Assessment	X			X	X
TMs (OHT & CWT delivery)	X	X	X	X	

7.1 Eligibility Determination

Subjects who express an interest in study participation will talk on the phone or meet in person with a RA or other appropriate study staff who will determine eligibility based on a series of eligibility questions (see Section 5). Those who are deemed eligible will continue with the consent process conducted by the RA.

7.2 Enrollment/Baseline

Subjects will be enrolled in the study after signing an electronic informed consent form and completing all baseline activities. For some participants, these activities will occur on the same day as eligibility determination and consent. For others, screening and review of consent procedures may take place over the phone (prior to their clinic appointment), and documentation of informed consent, the baseline questionnaire, and the oral assessment will occur at in person.

The following activities will occur at baseline:

- Informed consent
- Opt-in to Agile Health system
- Contact information and demographic information
- Completion of Baseline Questionnaire

- Conduct oral health assessment of child
- Randomize participant to OHT or CWT
- TM Intervention will begin after randomization

7.3 Follow-up

Follow-up assessments will occur approximately at 2, 4, 12 and 24- months following the baseline visit. For the 2 and 4 month survey, participants completing the survey within 14 days prior to or 31 days after the target date will be considered compliant with the study schedule. Oral health assessments of children and surveys at 12 and 24- months occurring within -14 days prior to or 60 days after the target follow-up date will be considered compliant with the study follow-up schedule. Surveys will be completed through electronic data capture, either in-person, on-line, or via weblink. If the person requests that the survey be conducted via interview, we will oblige and administer the survey verbally and the RA will input data directly into the electronic data capture system.

Baseline – 4 months:

The intervention will be delivered to caregivers via daily TM for 4- months. However, there are no study visits associated with the intervention delivery.

2 and 4 month assessments (-14 days/+ 31 days):

- Record adverse events as reported by subject or observed by investigator.
- Self-report questionnaire

12 month assessment (12 months -14 days/+ 60days)

- Record adverse events as reported by subject or observed by investigator.
- Self-report questionnaire
- Conduct oral health assessment of child

7.4 Final Study Visit (24 months -14 days + 60 days)

The final study visit will occur at 24 months after baseline, within 7 days prior to or 31 days after the target date. The final visit will occur sooner than 24 months if a family has moved or withdrawn from the study and we conduct the final clinical visit.

At the final study visit the study staff will:

- Record adverse events as reported by subject or observed by investigator.
- Conduct oral health assessment of the child
- Self-report questionnaire

- Provide final instructions to subject including how to follow-up of ongoing adverse events

7.5 Early Termination Visit

If early termination occurs, we will make a minimum of 5 attempts to contact the caregiver in order to complete the oral health clinical assessment and questionnaire.

7.6 Unscheduled Visit

An unscheduled visit would be considered to occur if a study subject was in contact with a member of the study team regarding oral knowledge, attitudes or practices if the contact was not: 1. part of normal texting procedures or 2. related to the occurrence of adverse events or unanticipated problems. All unscheduled visits will be documented via a note to file and included in the participants' research record.

8 STUDY PROCEDURES /EVALUATIONS

8.1 Study Procedures/Evaluations

Screening Administration:

Trained study staff, usually an RA, will screen individuals for eligibility as described above and in Section 5. At screening, all participants will be administered the "AUDIT-C," which is a 3-item alcohol screen (range 0-12) that identifies persons who are hazardous drinkers or have active alcohol use disorders (Hodgson et al., 2003). In men, a score of 4 or more is considered positive, and in women a score of 3 or more is considered positive. For drug misuse screening (Smith et al., 2010), we will ask if they have, in the last two weeks, used any of the following medicines on their own, without a doctor's prescription, in greater amounts or longer than prescribed (e.g., opioid pain killers (like Vicodin), stimulants, (e.g. Ritalin or Adderall), sedatives or tranquilizers (e.g. Valium) or drugs like cocaine or crack, club drugs (e.g. ecstasy), hallucinogens (e.g. LSD), heroin, inhalants or solvents (e.g. glue), or methamphetamines (e.g. speed). Participants will answer yes or no to this single question with no further questions about which drugs they have used. Participants who answer 'yes' to this single question will not be enrolled in the study.

Additionally, participants will be asked to self-report diagnosis of serious mental illness (Bipolar Disorder, Mania, Manic-depressive disorder, Schizophrenia, Psychosis). At screening, all participants will be asked "Have you ever been diagnosed with a mental illness?" and if so, which ones. If the participant reports that he or she has been diagnosed with one of the mental illnesses listed above, they will be excluded.

Study data collected at screening includes documentation of all eligibility and exclusion criteria. These data will be recorded using EDC.

Questionnaire Administration:

At baseline, after participants complete the informed consent process, study staff will verbally collect contact information and demographic information on the Contact Information eCRF. Study staff will enter information directly into REDCAP to ensure that the child, for whom the informed consent had been collected, matches the child's information for the text message program (i.e., child's first name, child's gender, and child's date of birth.)

All study assessment points include completion of questionnaires by parent participants, to assess each of the outcome measures described in Section 3. The participant baseline questionnaire will be conducted in person using EDC at the pediatric clinic. However, when that is not possible, participants will have the ability to complete the questionnaire via a secure website on their own laptop or other device or via smartphone or over the phone with the study team assistance.

2 month assessment: a shortened version of the survey, which primarily focuses on collection of mediators, will be utilized to assess intervention processes and impact while being sensitive to respondent burden and the impact of respondent burden on retention.

4 month assessment: Includes a collection of mediators, secondary outcomes and program satisfaction data.

Oral Health Assessments (Baseline, 12 and 24 months): The OHDC Modified ICDAS protocol that was developed as a collaborative effort of the CC and the study PIs will be utilized to collect caries outcome data. This standardized protocol will be used to assess the presence or absence of dental caries at the tooth surface level by a trained, calibrated clinical examiner blinded to treatment condition. Dental caries assessment will occur at baseline and then annually and in most cases occur at the pediatric clinic. If the parent cannot bring the child to the pediatric clinic, the clinical examiner will utilize a suitable alternative setting to conduct the visits, such as the participants' home.

Referral Procedures

Referral codes

- 1 - There is no visible decay (Regular dental exams are recommended)
- 2 - Early dental care is needed (< 5 decayed tooth surfaces)
- 3 - Treatment is needed soon (5 or more decayed surfaces)
- 4 - Treatment is urgently needed (Pain is reported or infection is present)

Process for referrals

A report card indicating each child's oral health status and need for dental treatment will be given to caregivers immediately following the child's clinical oral health assessment. The report card includes the phone number of the RA and a referral list of local providers to assist the caregiver in locating dental treatment for their child.

For children with a referral code of 4, one week following the oral health assessment, the caregiver will be contacted to ensure that treatment was obtained. If care was not provided, the caller will offer to make an appointment for the child.

Medical Emergency Protocol

Clinical Examiners will:

- Be equipped with medical emergency kits containing first aid supplies, epi pens, glucose gel, etc.

- Be Certified in Cardiopulmonary Resuscitation

- Be aware of emergency protocols at each pediatric clinic including emergency exits and location of telephones

Intervention Dose and Engagement:

Agile Health will send regularly scheduled reports which will include data regarding:

- Number of texts sent
- Percent of texts responded to
- Number prompted responses
- Use of program features
- Topics chosen
- Goals set/completed
- Ratings of TMs and program satisfaction

Budget Impact Evaluation:

We will survey IT specialists at all our sites as well as technical experts in industry to estimate interface and application costs of two different integration strategies involving the incorporation of the OHT intervention into routine clinical care: 1) a fully-automated strategy that involves an EHR prompt to providers recommending that they 'prescribe' the program to their patients and the system automatically enrolls the patient via an EHR to iSMILE and where a summary is returned to the EHR for guidance at future appointments; 2) a more labor intensive strategy but less technologically complex approach whereby the provider creates 'an order' in the EHR and a printout is initiated for the patient and the patient signs themselves up. Interviews with decision makers from our currently targeted sites will be used to explore the barriers and facilitators to the intervention implementation and adoption.

8.2 Laboratory Procedures/Evaluations

No laboratory procedures or evaluations will be performed as part of this study.

9 ASSESSMENT OF SAFETY

9.1 Specification of Safety Parameters

Safety monitoring for this study will focus on unanticipated problems involving risks to participants, including unanticipated problems that meet the definition of a serious adverse event.

9.1.1 Unanticipated Problems

Unanticipated problems involving risk to human subjects will be recorded and reported throughout the entire study.

The Office for Human Research Protections (OHRP) considers unanticipated problems involving risks to subjects or others to include, in general, any incident, experience, or outcome that meets **all** of the following criteria:

- unexpected in terms of nature, severity, or frequency given (a) the research procedures that are described in the protocol-related documents, such as the IRB-approved research protocol and informed consent document; and (b) the characteristics of the subject population being studied;
- related or possibly related to participation in the research ("possibly related" means there is a reasonable possibility that the incident, experience, or outcome may have been caused by the procedures involved in the research); and
- suggests that the research places subjects or others at a greater risk of harm (including physical, psychological, economic, or social harm) than was previously known or recognized.

9.1.2 Adverse Events

Adverse events that are plausibly related to the study will be recorded and reported throughout the entire study.

An adverse event is any untoward or unfavorable medical occurrence in a human subject, including any abnormal sign (for example, abnormal physical exam or laboratory finding), symptom, or disease, temporally associated with the subject's participation in the research and considered related to the subject's participation in the research.

9.1.3 Serious Adverse Events

Serious adverse events will be recorded and reported throughout the entire study.

A serious adverse event (SAE) is one that meets one or more of the following criteria:

- Results in death
- Is life-threatening (places the subject at immediate risk of death from the event as it occurred)

- Results in inpatient hospitalization or prolongation of existing hospitalization
- Results in a persistent or significant disability or incapacity
- Results in a congenital anomaly or birth defect
- An important medical event that may not result in death, be life threatening, or require hospitalization may be considered an SAE when, based upon appropriate medical judgment, the event may jeopardize the subject and may require medical or surgical intervention to prevent one of the outcomes listed in this definition.

9.2 Time Period and Frequency for Event Assessment and Follow-Up

AEs, UPs and SAEs will be recorded in the data collection system throughout the study. Events will be followed for outcome information until resolution or stabilization.

9.3 Characteristics of an Adverse Event

9.3.1 Relationship to Study Intervention

To assess relationship of an event to study intervention, the following guidelines are used by the Co-PIs:

1. Related (Possible, Probable, Definite)
   a. The event is known to occur with the study intervention.
   b. There is a temporal relationship between the intervention and event onset.
   c. The event abates when the intervention is discontinued.
   d. The event reappears upon a re-challenge with the intervention.
2. Not Related (Unlikely, Not Related)
   a. There is no temporal relationship between the intervention and event onset.
   b. An alternate etiology has been established.

9.3.2 Expectedness of SAEs

The study PIs will be responsible for determining whether an SAE is expected or unexpected. An adverse event will be considered unexpected if the nature, severity, or frequency of the event is not consistent with the risk information previously described for the intervention.

9.3.3 Severity of Event

The following scale will be used by the Co-PIs to grade adverse events:

1. Mild: no intervention required; no impact on activities of daily living (ADL)

2. Moderate: minimal, local, or non-invasive intervention indicated; moderate impact on ADL

3. Severe: significant symptoms requiring invasive intervention; subject seeks medical attention, needs major assistance with ADL

9.4 Reporting Procedures

9.4.1 Unanticipated Problem Reporting to IRB and NIDCR

Incidents or events that meet the OHRP criteria for unanticipated problems require the creation and completion of an unanticipated problem report form by the Co-PIs. The report will include the following information when reporting an adverse event, or any other incident, experience, or outcome as an unanticipated problem to the IRB:

- appropriate identifying information for the research protocol, such as the title, investigator's name, and the IRB project number;
- a detailed description of the adverse event, incident, experience, or outcome;
- an explanation of the basis for determining that the adverse event, incident, experience, or outcome represents an unanticipated problem;
- a description of any changes to the protocol or other corrective actions that have been taken or are proposed in response to the unanticipated problem.

To satisfy the requirement for prompt reporting, unanticipated problems will be reported using the following timeline:

- Unanticipated problems that are serious adverse events will be reported to the IRB and to NIDCR within 24 hours if death or a life-threatening event or within 72 hours for all other serious adverse events.
- Any other unanticipated problem will be reported to the IRB and to NIDCR within 7 days of the investigator becoming aware of the problem.
- All unanticipated problems should be reported to appropriate institutional officials (as required by an institution's written reporting procedures). The IRB will then report to the supporting agency head (or designee), and OHRP within one month of the IRB's receipt of the report of the problem from the investigator.

All unanticipated problems will be reported to NIDCR's centralized reporting system via Rho Product Safety:

- Product Safety Fax Line (US): 1-888-746-3293
- Product Safety Fax Line (International): 919-287-3998
- Product Safety Email: rho_productsafety@rhoworld.com

General questions about SAE reporting can be directed to the Rho Product Safety Help Line (available 8:00AM – 5:00PM Eastern Time):

- US: 1-888-746-7231
- International: 919-595-6486

9.4.2 Serious Adverse Event Reporting to NIDCR

For any AE meeting the specified Serious Adverse Event criteria, the CO-PIs or their designee will submit an SAE form to NIDCR's centralized safety system via Rho Product Safety. This report may be sent by fax or email. Once submitted, Rho Product Safety will send a confirmation email to the investigator within 1 business day. The investigator should contact Rho Product Safety if this confirmation is not received. This process applies to both initial and follow-up SAE reports.

SAE Reporting Contact Information:

- Product Safety Fax Line (US): 1-888-746-3293
- Product Safety Fax Line (International): 919-287-3998
- Product Safety Email: rho_productsafety@rhoworld.com

General questions about SAE reporting can be directed to the Rho Product Safety Help Line (available 8:00AM – 5:00PM Eastern Time):

- US: 1-888-746-7231
- International: 919-595-6486

The study clinician will complete a Serious Adverse Event Form and submit via fax or email within the following timelines:

- All deaths and immediately life-threatening events, whether related or unrelated, will be recorded on the Serious Adverse Event Form and submitted to Product Safety within 24 hours of site awareness.
- Serious adverse events other than death and immediately life-threatening events, regardless of relationship, will be reported by fax within 72 hours of site awareness.

All SAEs will be followed until resolution or stabilization.

10 STUDY OVERSIGHT

Study oversight will be under the direction of the study PIs, Dr. Borrelli (lead administrative and fiscal PI), Dr. Henshaw, and the NIDCR. The study PIs will have weekly scheduled meetings to review study progress and monthly meetings with NIDCR and the DCC.

In addition to the PI's responsibility for oversight, study oversight will be under the direction of a Data and Safety Monitoring Board (DSMB) composed of members with appropriate clinical, statistical, scientific, and ethical expertise. The NIDCR will appoint the Board. The DSMB will meet annually to assess safety and efficacy data, study progress, and data integrity for the study. If concerns arise, more frequent meetings may be held. The DSMB will operate under the rules of an NIDCR-approved charter that will be approved at the organizational meeting of the DSMB. The DSMB will provide recommendations to the NIDCR.

11 CLINICAL SITE MONITORING

Clinical site monitoring is conducted to ensure that the rights of human subjects are protected, that the study is implemented in accordance with the protocol and/or other operating procedures, and that the quality and integrity of study data and data collection methods are maintained. Monitoring for this study will be performed by NIDCR's Clinical Research Operations and Management Support (CROMS) contractor. The monitor will evaluate study processes and documentation based on NIDCR standards and the International Conference on Harmonisation (ICH), E6: Good Clinical Practice guidelines (GCP).

Details of clinical site monitoring will be documented in a Clinical Monitoring Plan (CMP) developed by the CROMS contractor, in collaboration with the NIDCR Office of Clinical Trials and Operations Management (OCTOM) and the NIDCR Program Official. The CMP will specify the frequency of monitoring, monitoring procedures, the level of clinical site monitoring activities (e.g., the percentage of subject data to be reviewed), and the distribution of monitoring reports. Some monitoring activities may be performed remotely, while others may take place at the study site(s). Staff from the CROMS contractor will conduct monitoring activities and provide reports of the findings and associated action items in accordance with the details described in the CMP. Documentation of monitoring activities and findings will be provided to the site study team, the study PIs, OCTOM, and the NIDCR. The NIDCR reserves the right to conduct independent audits as necessary.

12 STATISTICAL CONSIDERATIONS

12.1 Study Hypotheses

Primary hypothesis 1: Those receiving OHT will have lower 24 month incidence of any new caries, compared to those receiving CWT.

Primary hypothesis 2: The OHT effect on new caries at 24 months will be mediated through Social Cognitive Theory constructs (self-efficacy, motivation, and outcome expectations). We will examine indirect effects of OHT through self-efficacy, motivation, and outcome expectations at 2 months, to potentially increased motivation and oral health behavior at 4 months (end of intervention) to new caries at 12 and 24 months. We will also examine indirect effects of OHT through self-efficacy, motivation, and outcome expectations at 4 months (end of treatment), to potentially increased motivation and oral health behavior at 12 months and to new caries at 12 and 24 months.

Secondary hypothesis 1: We hypothesize that the total cost of OHT implementation will be more cost-effective than standard of care (e.g., oral health anticipatory guidance).

Secondary hypothesis 2: Those receiving OHT will have better pediatric oral health behaviors (tooth brushing, diet, sugar-sweetened beverages, fluoride toothpaste use, preventive dental visits, and oral assessment by parents) at 4 months (end of intervention), 12 months, and 24 months, compared to those receiving CWT.

Secondary hypothesis 3: Those receiving OHT will have better oral health behaviors (tooth brushing, dental visits), oral health knowledge, and attitudes at 4 months (end of intervention), 12 months, and 24 months, compared to those receiving CWT.

Exploratory Analyses: These analyses will explore whether the effect of OHT vs. CWT on new caries at 24 months is greater in subpopulations defined by race/ethnicity, insurance coverage, parent education level, child's prior caries experience, parent depressed mood, and intervention dose/engagement.

12.2 Sample Size Considerations

Primary sample size considerations focused on primary hypothesis 1, testing for a difference in the percentage of children with new caries at 24 months in those randomized to OHT vs. CWT. Sample size was calculated for the chi-square test comparing these two independent percentages, testing at the two-tailed alpha (Type I error rate) of 0.05 level and achieving 80% power (0.20 Type II error rate).

No interim analyses are planned.

Power calculations assumed an underlying new caries incidence at 24 months of 23.8% in the CWT group vs. 14.3% in the OHT group (corresponding to an odds ratio of 0.53; justification given below).

Based on our experience in the pilot study and our previous studies (e.g., the recently completed OHAPH trial), we anticipate examining 70% of enrolled parent/child dyads at 24

months. We do not anticipate missing data other than that due to loss-to-follow-up. We will attempt to follow and include all parents in the analysis regardless of their compliance with the study protocol and level of engagement with the OHT or CWT intervention ('intent to treat'). Parents lost to follow-up will not be replaced. Participants, can, however, become ineligible after randomization (and not evaluable), for example, if a parent loses custody of the child. Participants who become ineligible after randomization will be replaced if the ineligibility occurs while the study is still recruiting. If they become ineligible after the study recruitment period, their data will not be included in analyses.

Given the above scenario, an analysis sample of n=600 parent/child dyads (n=300 in each study arm) gives 84% power of detecting a significant decrease in caries incidence in the OHT vs. CWT group. This requires an enrollment sample of n=850 (n=425 in each study arm) to account for loss-to-follow-up.

If the underlying incidence of ECC is higher than the assumed 23.8% in the control CWT group, we will have greater power of detecting a difference between arms corresponding to an odds ratio of 0.53. For example, for an underlying incidence of ECC in the CWT group of 30%, the OR=0.53 corresponds to a difference in incidence of ECC of 18.6% vs 30% and a power of 90%. If the underlying incidence of ECC is lower in the CWT group, we will have lower power of detecting the OHT effect. For example, for an underlying incidence of ECC in the CWT group of 20%, the OR=0.53 corresponds to a difference in incidence of 11.8% vs 20% and a power of 0.78. If the underlying intervention effect is weaker than assumed, we will have lower power of detecting an effect. For example, with an incidence of ECC in the CWT group of 23.8% and an intervention effect corresponding to an odds ratio of 0.60 (incidence of 15.8% vs 23.8%) power would be 69%.

Power under different incidence levels and intervention effects, analysis samples of n=300 per study arm. The first row corresponds to our primary anticipated effect

OHT Effect Odds Ratio	ECC Incidence in CWT Group	ECC Incidence in OHT Group	Statistical Power
0.53	23.8%	14.3%	0.84
0.53	30.0%	18.6%	0.90
0.53	20.0%	11.8%	0.79
0.60	23.8%	15.8%	0.69
0.60	30.0%	20.4%	0.77

<u>Justification of the anticipated OHT vs. CWT effects</u>: Data from the behavioral interventions delivered by Kressin et al. (2009) and Weinstein et al. (2004) was used to calculate sample size and power because the Kressin oral health data was collected at the largest site in our study (BMC) and another pediatric clinic located within a teaching hospital 1 mile away (with a population comparable to that at our CHCs). In the study by Kressin, caries incidence in the control site was 31.7%; however, participants did not regularly receive fluoride varnish (FV), a known preventive measure which reduces caries risk. The application of FV is inconsistent and infrequent in our targeted CHCs, but if we conservatively assume that all child participants will receive the recommended 4 applications/year, then we can assume that the incidence of caries

among controls will be reduced an additional 25% (Weinstein et al, 2004) or from 31.7% to **23.8%.** Kressin et al. (2009) found a 44.2% reduction in caries incidence due to the brief patient centered counseling intervention during the pediatric well child visit. If we assume a similar effect from the daily text messaging intervention, we would expect a **13.3%** incidence of caries in the intervention group compared to 23.8% incidence in the control group (accounting for possible FV effect). To be slightly more conservative, we can use the 40% reduction in caries incidence found by Weinstein et al in his intervention and in the Marinho et al., 2013 Cochrane review. This would result in **14.3%** caries incidence for the intervention group. In this scenario, we would need 300 parent-child dyads at our 24 month follow-up to have an estimated 84% power (2 tailed) to detect a 9.5% difference in caries incidence between groups with alpha=0.05 and according to the standard chi-square test.

Our second primary hypothesis will examine the mechanism of the OHT effect, examining mediation through Social Cognitive Theory constructs of self-efficacy, motivation, and outcome expectations. Statistical power for tests of indirect effects of OHT through a mediator will be calculated using the joint effects approach; there is 80% power of detecting an indirect effect if the power of showing an association between OHT and the mediator, and an association between the mediator and 24 month caries incidence, are both 90%. We have 80% power of showing an indirect effect of a continuous mediator if intervention (OHT vs. CWT) explains 2% of the mediator (R^2=.02), and if a 1 standard deviation increase in the mediator leads to a 30% reduction in the odds of caries (an OR=0.70). This study was powered to detect an OHT effect in the overall sample. Secondary analyses examining whether the OHT effect varies across subpopulations defined by race/ethnicity, insurance coverage, maternal education level, or child's prior caries experience will have lower power. These analyses should therefore be considered exploratory.

12.3 Planned Interim Analyses (if applicable)

There is minimal risk associated with the interventions and no interim analysis are planned.

12.3.1 Safety Review

Safety concerns associated with this study are minimal and there are no plans to halt study enrollment in response to a Safety Review.

12.3.2 Efficacy Review

There is minimal risk associated with this study. No interim analyses are planned, and there are no plans to halt study enrollment in response to an efficacy review.

12.4 Final Analysis Plan

Overview of Analytic Plan: Primary analyses will focus on the comparison of those assigned to OHT vs. CWT on oral health and behavior outcomes using all available data (i.e., restricted to those followed) and, following the intent-to-treat principle, randomized subjects will be included in the analysis regardless of their compliance with the study protocol and engagement in the study interventions. While no differences between study arms are anticipated due to randomization, preliminary analyses will explore differences between arms and variables found to differ between groups (at the p<0.10 level) will be adjusted for in analysis. For dichotomous outcome variables (e.g., incidence of any new caries at 24 months; use of fluoride toothpaste) analyses will use logistic regression models, with intervention effects reported as odds ratios

with 95% confidence intervals. Count data (e.g., number of new caries at 24 months) will be analyzed through Poisson regression models, if the distributional assumptions of the Poisson model are met, or negative binomial or zero-inflated models if the outcome distribution better meets the assumptions of these models. Results will be reported as rate ratios with 95% confidence intervals. Continuous variables (e.g., number of tooth brushings per week) will be analyzed through mixed effects linear regression models. Data will also be analyzed by race and gender as specified in the Notice of Grant Award, although these analyses will be exploratory due to lack of power. If follow-up falls below the anticipated 70%, primary analyses will account for missing data through inverse probability weighting using propensity scores for missingness. If follow-up meets the anticipated 70%, inverse probability weighting may be used in sensitivity analyses addressing the impact of missing data.

The two-tailed alpha 0.05 level will be used to indicate statistical significance.

Primary aim 1: We will use logistic regression to compare the incidence of new caries in primary dentition (dmfs) over 24 months (no vs. any new caries) in OHT vs. CWT children, controlling for age, previous caries, and other relevant variables. Secondary analyses will also use logistic regression to compare OHT vs. CWT on the incidence of new caries in permanent dentition (DMFS) and mixed dentition (dmfs+DMFS). Secondary analyses of the number of new caries will be conducted using Poisson regression or related methods. Distributional assumptions of Poisson regression will be checked, and negative binomial regression models (for over-dispersion) and zero-inflated (negative binomial and Poisson) models (for excess zeros) will be considered if appropriate. Given that we are performing oral assessments at three time points (baseline, 12 and 24 months), we can also perform a tooth-level analysis of new caries in primary and permanent dentition, using mixed effects logistic regression models (Gibbons et al., 2010) to account for the multiple observations (teeth) per child.

Primary aim 2: Our second primary aim is to investigate the mechanisms of the OHT intervention effect on caries incidence (mediation analysis). Mediation analyses will examine indirect effects of OHT on caries incidence through constructs from Social Cognitive Theory (self-efficacy, motivation, and outcome expectations). We hypothesize that OHT will lead to improved self-efficacy, motivation, and outcome expectations, which in turn will be associated with better proximal outcomes (increased motivation and oral health behavior) at the end of treatment, 4 months. We further hypothesize that changes in mediators over the course of treatment will have a mediational effect on distal outcomes (change in oral health behaviors and lower caries incidence at 12 and 24 months). For descriptive analyses of mediation, we will take a causal steps approach and test individual mediators, as specified by MacKinnon and Fairchild (2007). Then, we will use the product of coefficients method, using bootstrapped confidence intervals (Shrout & Bolger, 2002) to account for the non-normality of the estimated indirect effect, which utilizes a multiple mediation model to simultaneously test the effects of potential mediators, and enabling the determination of the relative influence of each. We will also consider using a counterfactual approach (Valeri & Vanderweele, 2013) although it does not allow for time varying effects of mediators, and has not been previously used in oral health research like the MacKinnon procedure.

Secondary aims

Aim 1. We will conduct a Budget Impact Analysis (BIA) to assess the financial consequences of adoption, diffusion and sustainability of the OHT intervention. BIA will be undertaken from the perspective of the budget holder, who makes decisions regarding uptake of interventions. To do this, we will follow the best practice guidelines set forth by the International Society for Pharmacoeconomics and Outcomes Research (ISPOR) task force (Mauskopf et al., 2007). As such, we will identify detailed cost and claims data to assess the cost impact of providing the intervention and compare the clinical outcomes of the OHT intervention with the costs of achieving those outcomes, and conduct sensitivity analyses to present context specific scenarios for different situations (e.g., pediatric clinics that may have a lower/higher proportion of Medicaid patients). Sensitivity analysis will model two different strategies to incorporate the OHT intervention into routine clinical care: 1) a fully-automated strategy that involves an EHR prompt to providers recommending that they 'prescribe' the program to their patients and the system automatically enrolls the patient via an EHR to iSMILE and where a summary is returned to the EHR for guidance at future appointments; 2) a more labor intensive strategy but less technologically complex approach whereby the provider creates 'an order' in the EHR and a printout is initiated for the patient and the patient signs themselves up. We will be able to calculate the cost per caries prevented under each scenario. BIA is essential to helping potential diffusion targets to form business plans that determine the financial feasibility of the OHT intervention. We will survey IT specialists at all our sites as well as technical experts in industry to estimate interface and application costs. BIA is a useful approach to help stakeholder decision makers decide if this particular intervention is worth creating and sustaining beyond grant based startup funding. Policy decision makers will be able to plug in and alter assumptions as needed for their situations. We will explore whether or not it is possible to assess site specific effects using multilevel modeling (Charns et al., 2012). Interviews with decision makers from our currently targeted sites will be used to explore the barriers and facilitators to the intervention implementation and adoption. This will help develop plans for "implementation spread" to other sites that can be tailored to local requirements and needs.
Aims 2 and 3. We will compare changes in these behaviors over time in OHT vs. CWT using mixed effects logistic regression models (for dichotomous outcomes) and mixed effects linear regression models (for continuous outcomes). Changes in these behaviors from baseline to 2, 4, 12, and 24 months will be described through odds ratios and 95% confidence intervals for logistic models, and slopes for linear models. Differences in changes over time for OHT vs. CWT will be tested through group by time interaction terms.
Aim 4: To identify subpopulations for whom the intervention has the greatest effect, we will analyze moderators; e.g., race/ethnicity, insurance coverage, parent educational level, and child's prior caries experience. This aim focuses on moderator effects (also known as effect modification) for incident caries over 24 months. We will use logistic regression models with multiplicative interaction terms (treatment group x moderator) to compare the efficacy of OHT across subpopulations.

17. Missing data: We will gather follow-up information and reasons for dropout regardless of treatment completion, censoring at the point of loss. We will examine the extent of drop-out, and identify characteristics associated with drop-out through logistic regression. Given drop-out does not exceed the expected rate of 30%, primary analyses will be based on available data. Secondary analyses will account for missing data and explore the potential impact of drop-out on study findings. We will describe the robustness of our findings with respect to missing data using inverse probability weighting. This approach to handling missing data has two steps: 1) develop propensity scores for missing data, which involves using logistic regression to model the probability of being followed to the 24 month visit as a function of baseline variables, including history of caries; and 2) using the inverse of the propensity scores (predicted probabilities from the first step) to serve as weights in our logistic regression model of new

primary teeth caries. Provided the data are missing at random (MAR), that is, the probability of missingness can be fully explained by observable data, this approach produces asymptotically unbiased estimates. If there is sufficient missing data, and a concern that data may be missing not at random, we will also consider pattern mixture models to perform a sensitivity analysis of the potential impact of missingness.

13 SOURCE DOCUMENTS AND ACCESS TO SOURCE DATA/DOCUMENTS

Study staff will maintain appropriate medical and research records for this study, in compliance with ICH E6, Section 4.9 and regulatory and institutional requirements for the protection of confidentiality of subjects. Study staff will permit authorized representatives of NIDCR and regulatory agencies to examine (and when required by applicable law, to copy) research records for the purposes of quality assurance reviews, audits, and evaluation of the study safety, progress and data validity.

Access to source data and documents is controlled through strict physical and electronic controls. Staff access permissions will be set according to job description activities.

Source documents will include informed consent forms completed electronically in REDCap, a secure web-based application. Paper consent forms will be available as an alternative as necessary. This information will be kept in locked file cabinets, within a locked office within GSDM's research offices at 560 Harrison Avenue and only study staff will have keys. With the exception of the data collected through caregiver TMs, the remainder of the source documents will be eCRFs. These include iSMILE screener, iSMILE questionnaire, and caries assessment data capture systems, such as REDCAP. The iSMILE specific eCRFs were developed in concert with the CC as part of the qualitative and pilot phase. The eCRFs in CARIN were developed by UCSF's CC and successfully utilized by the Co-PIs during a previous RCT funded by the NIDCR (Oral Health Advocates in Public Housing).

Agile Health (AH) is our technology partner. The AH platform is the primary source of TM data. The platform, which is seen through a web-based user interface dashboard, requires permissions to access as well as a login. The dashboard allows our team to monitor incoming and outgoing messages in real-time, set personalization without help from a programmer, and respond to participant inactivity. The AH platform is HIPAA-compliant, with all PHI / PII data encrypted in transit and at rest, and messages are delivered through a secure gateway in an encrypted format.

The iSMILE specific eCRFs for this trial will be housed at the CC. Server racks are bolted to the foundation beneath the concrete floor for earthquake preparedness. Physical access is strictly controlled. Electronic access is protected with 2 state-of-the-art hardware firewalls, strong password protection, and automatic system log-outs. All data are encrypted and backed up onto servers daily. Encrypted backup tapes are stored off-site weekly and monthly by a leading vendor of storage services (Iron Mountain). Disaster recovery drills will be conducted monthly.

14 QUALITY CONTROL AND QUALITY ASSURANCE

Quality Management is the overall process of establishing and ensuring the quality of processes, data, and documentation associated with clinical research activities. It encompasses both quality control (QC), and quality assurance (QA) activities.

Ultimate responsibility for implementation and maintaining quality assurance and quality control systems with written operating procedures to ensure that the trial is conducted and data are generated, documented and reported in compliance with the protocol resides with the principal investigators of the study. The project director, CC, and AH will provide regular reports on the fidelity and administration of the intervention (listed in section 15.4) to the Co-PIs. These processes are outlined in the study's Quality Management Plan.

The NIDCR Oral Health Disparities in Children (OHDC) Consortium's Coordinating Center (CC) has developed the Caries and Plaque Screening Examination Training and Calibration for Oral Health Disparities in Children Consortium" and annually will conduct in person calibration sessions that utilize live patients and adhere to all CP guidelines. All of our clinical examiners will attend a calibration session each year and attain competency, based on protocol standards, prior to collecting any clinical oral health data. All calibration sessions will follow the protocol outlined in "Caries and Plaque Screening Examination Training and Calibration for Oral Health Disparities in Children Consortium." If for some reason a secondary examiner cannot attend the annual CC sessions (for example – a clinical examiner is hired mid-year), he/she will be trained and calibrated using the same procedures by the CC, and compared to the Center's gold standard examiner.

15 ETHICS/PROTECTION OF HUMAN SUBJECTS

15.1 Ethical Standard

The investigator will ensure that this study is conducted in full conformity with the principles set forth in The Belmont Report: Ethical Principles and Guidelines for the Protection of Human Subjects of Research, as drafted by the US National Commission for the Protection of Human Subjects of Biomedical and Behavioral Research (April 18, 1979) and codified in 45 CFR Part 46 and/or the ICH E6.

15.2 Institutional Review Board

The protocol, informed consent form(s), recruitment materials, and all subject materials will be submitted to the BUMC IRB for review and approval. Approval of both the protocol and the consent form will be obtained before any subject is enrolled. Any amendment to the protocol will require review and approval by the IRB before the changes are implemented in the study.

15.3 Informed Consent Process

Informed consent is a process that is initiated prior to the individual participating in the study and continues throughout study participation.

Prior to screening, we will obtain brief verbal consent to be screened. If screening takes place over the phone and the participant is eligible, we will retain their data and their identifiable contact information and the link between the two. Thus, we will also request a waiver of documentation of consent. If the screening takes place over the phone and the participant is ineligible, we will retain their non-identifiable screening information and destroy their contact information and any link between their contact information and screening data. In some cases, the baseline survey may be conducted over the phone or via online web link, prior to the participant arriving at the clinic. Thus, the waiver of documentation of consent will also be needed for this activity. If the baseline survey is performed at the clinic, the participants will complete written informed consent electronically at that time. Paper consent forms will be utilized in the event that the electronic consent fails. If the baseline survey is performed over the phone, informed consent will be obtained in writing prior to the oral assessment.

Extensive discussion of risks and possible benefits of study participation will be provided to participants and their families, if applicable. A paper copy of the consent form describing in detail the study procedures and risks will be given to the participant. Consent forms will be IRB-approved, and the participant is required to read and review the document or have the document read to him or her. The investigator or designee will explain the research study to the participant and answer any questions that may arise. Participants will be given the opportunity to discuss the study with their surrogates or think about it prior to agreeing to participate. They may withdraw consent at any time throughout the course of the study. A copy of the informed consent document will be given to participants for their records. The rights and welfare of the participants will be protected by emphasizing that the quality of their clinical care or their child's care will not be adversely affected if they decline to participate in this study.

The consent process will be documented in the research record.

Translation of the consent form into Spanish will be reviewed and approved by the IRB for non-English speaking subjects. The informed consent process will remain the same but will be conducted in the subject's preferred language by study staff who are bilingual in English and Spanish.

At the time of the oral health assessment, the verbal dissent of a child will be honored and those activities will not be completed.

RAs, or research study staff members conducting enrollment, will obtain informed consent. All informed consent procedures will be done in a private location and all potential participants will be given sufficient time to consider their choice to participate or not.

The IRB approved informed consent will be available to potential participants electronically through REDCap. The IRB approved informed consent describing in detail the study, including both procedures and risks, will be given to the potential participant in their preferred language (English or Spanish). The potential participant is required to read and review the informed consent document.

The RA or qualified study staff member will verbally review the informed consent in REDCap with the individual to ensure that the potential participant understands the study. The study staff member obtaining informed consent will also explain that the parent must consent to their child receiving a baseline, 12, and 24 month oral health assessment as part of required study enrollment. Additionally, the RA or study staff member obtaining consent will explain that the participant will be randomly enrolled into one of two different text message programs, making clear that the one that they are assigned to will be determined completely by chance. The RA or study staff member obtaining consent will answer any questions that may arise.

For eligible participants screened and enrolled over the phone, participants may either have the informed consent procedure done at the time of their clinic appointment, or the informed consent will be done prior to their clinic appointment with a waiver of documentation of consent. In the latter case, if the participant is willing and able to complete the baseline questionnaire before the clinic visit, they will be sent a web link to the questionnaire. However, participants will not be able to access or complete the Baseline questionnaire unless they click "agree" to the consent – this will be recorded in REDCap. When they arrive at their clinic appointment, we will obtain electronic written informed consent in REDCap at that time. Another scenario is a participant who is screened eligible over the phone and would rather complete the baseline questionnaire via phone interview, rather than web link. In this case, the research assistant will read the entire consent form to the prospective participant and obtain consent to administer the questionnaire prior to any study activity. As with the first scenario, when they arrive at the clinic appointment, we will obtain electronic written consent in REDCap at that time. These procedures will allow participants the option of completing the Baseline measure prior to their clinic appointment, thus decreasing participant burden during the clinic visit. It is required that electronic written informed consent be collected in REDCap at the time of the in-person baseline oral health assessment.

A copy of all signed informed consent documents will be given to subjects for their records and a copy will be retained for the research record. A copy of the electronic informed consent can either be e-mailed to the participant, or if preferred, the participant can be given a paper copy of the informed consent.

15.4 Exclusion of Women, Minorities, and Children (Special Populations)

This study does not exclude women, minorities or children from participating.

15.5 Subject Confidentiality

Subject confidentiality is strictly held in trust by the investigators, study staff, and the funding agency and their agents.

The study protocol, documentation, data, and all other information generated will be held in strict confidence. No information concerning the study or the data will be released to any unauthorized third party without prior written approval of the funding agency.

Any authorized representatives of the funding agency, including the study monitor, may inspect all study documents and records required to be maintained by the investigator, including but not limited to, medical records (office, clinic, or hospital) for the study subjects. The clinical study site will permit access to such records.

15.6 Future Use of Stored Specimens and Other Identifiable Data

This study will not be collecting or storing specimens.

16 DATA HANDLING AND RECORD KEEPING

The Co-PIs are responsible for ensuring the accuracy, completeness, legibility, and timeliness of the data reported. All source documents should be completed in accordance with the principles of good clinical practice in order to ensure accurate interpretation of data as detailed in MOP. The Co-PIs will maintain case histories of study subjects, including accurate case report forms (CRFs), and all source documentation.

16.1 Data Management Responsibilities

Data collection and accurate documentation are the responsibility of the study staff under the supervision of the Co-PIs. All source documents and laboratory reports must be reviewed by the study team and data entry staff, who will ensure that they are accurate and complete. Unanticipated problems and adverse events must be reviewed by the Co-PIs or their designee. The CC will provide the study staff with access to the reports listed in section 16.4 that will allow the study team to monitor data quality.

The CC will provide oversight by running reports that show unresolved data discrepancies that have not been addressed by the study team.

16.2 Data Capture Methods

Data will be collected using several methods. Signed and dated consent forms will be completed electronically by the participants through REDCap, a secure, HIPAA-compliant web-based application. Paper consent forms will be utilized in the event that electronic capture is unsuccessful and will be stored at the BUGSDM offices under secured conditions. Screening and questionnaire data will be collected through a secure web-based EDC for direct data entry. REDCap will be used to collect data during the oral assessment. For each eCRF, a paper form will also be developed and utilized in the rare event that the electronic system is not available. All paper forms will be manually entered into the database and the paper forms stored in the subjects' research record. Data will also be captured through participants' responses to TMs during intervention delivery. Data collected from research participants will be stored in a secured, password protected computer file that is separate from identifiers. Any paper data will be placed in a locked file cabinet. A file will be maintained that associates the subject name with that subject's study identification number. This file will be kept in a secure, password-protected file, separate from the actual study data (e.g. screener and survey data).

16.3 Types of Data

Screening and questionnaire data are self-reported from participants and include but are not limited to: sociodemographic data; parental oral health knowledge, attitudes, and behaviors; psychological mediators/moderators; other health and risk factors; dental utilization data. This data is entered into REDCAP or other electronic data capture software. Some sociodemographic data required for enrollment into the text message system will be collected verbally by study staff using a Contact Information eCRF. Study staff will collect participant information prior to beginning the self-report baseline questionnaire. Information from participants include but are not limited to: child's first name or nickname; child's gender; child's date of birth. Study staff will enter data directly into REDCAP or other electronic data capture software. Surface level caries data is collected from the children's oral health assessment conducted by the calibrated dental examiner and recorded in REDCAP or other CC approved

electronic data capture software. TM data will include information on caries risk factors, parent knowledge, attitude and behaviors.

16.4 Schedule and Content of Reports

Report Name	Description/Purpose	Frequency	Recipients/ Users	Method for Provision
**EXAMPLES of reports that will be used by iSMILE**				
Accrual and Attended Visits Summary	The report provides up-to-date, pertinent information on the UH2/UH3 accrual and attended follow-up visits.<br><br>The PDF report is produced using SAS with data exported from the EDC.	Monthly	The CC Project Manager sends out the Accrual And Attended Visits Summary Report to everyone who attends the CC-NIDCR-UH2/UH3 Project Staff Monthly meeting.	Emailed attachment and upload to the UH2/UH3 SharePoint site.<br><br>The PDF report is produced using SAS with data exported from the EDC system.
Caries Exam Report	**Missing Caries Exams:** This tab lists participants who have an occurred caries exam visit but are missing the corresponding caries exam.<br><br>**Study identification numbers Missing Surface Data:** This tab lists all randomized participants who have missing tooth surface scores.<br><br>**Calendar Visit Count:** This tab shows the total number of caries exams performed during each study visit (e.g., baseline, 12 month, etc.).<br><br>**Tooth Status Check:** This tab shows any participant who has a tooth status that has changed in an unexpected way from one exam to the next observed exam. (e.g. If Tooth 16's status changes from present in the 18-month visit to unerupted in the 24-month visit).<br><br>For a given participant/visit combination, all teeth with exceptions are presented in a single row. If a participant misses a visit, for example, the 18 Month visit, but then receives an exam at 15 and 21 Months, then each tooth status	Monthly	The CC Project Manager sends out the Caries Report to everyone who attends the CC-NIDCR-Project Staff Monthly meeting.	Emailed attachment and upload to the UH2/UH3 SharePoint site.<br><br>The Excel report is produced by SAS with data exported from the EDC system.

	transition between 15 and 21 Months will be analyzed. Status transitions that are expected or unchanged are not shown in this tab.<br><br>**Surface Status Check:** This tab shows any participant/surface combination whose surface status has changed in an unexpected way from one visit to the next. (e.g. If Tooth 51's lingual surface status changes from fillingNonAmalgam in the 21-month visit to sound in the 24-month visit). Status transitions that are expected or unchanged are not shown in this tab.<br><br>**Surface Agreement Overall:** This tab contains a complete mapping of surface status changes from one exam to the next consecutive, occurred exam. (e.g. the surface status of a tooth changes from decay Cavitated to missing Caries). The earlier status is shown in the rows, whereas the latter status is shown in the columns. Perfect agreement, in a hypothetical scenario where no status changes occur, would be indicated by all non zero counts along the diagonal, and all off-diagonal counts equal to zero.<br><br>**Surface Agreement Overall 2:** This tab shows the Test of Symmetry in the overall surface agreement. *This tab can be ignored for routine operational purposes*.<br><br>**Surface Agreement Overall 3:** This tab gives some additional Kappa statistics. *This tab can be ignored for routine operational purposes*.<br><br>**Surface Agreement, Same Examiner:** This tab presents output similar to that of the Overall Surface Agreement tab, except that only the exams conducted by the same examiner are presented. So, if a Baseline and 3 Month exam			

	were conducted by Examiner #1, and the 6 Month exam was conducted by Examiner #2, and the 9 and 12 Month exams were conducted by Examiner #1, then only the Baseline-3 Month pair, and the 9-12 Month pair data would be shown. All mixed-examiner consecutive combinations are not included in these totals.<br><br>**Surface Agreement, Same Examiner 2:** This tab shows the Test of Symmetry in the overall surface agreement. *This tab can be ignored for routine operational purposes*.<br><br>**Surface Agreement, Reversals:** This tab uses a subset of the data in the Surface Agreement Overall tab. Only those surface transitions that represent an unlikely transition, e.g., from Missing to Sound, are represented here. The cell counts in this tab are identical to the cell counts in the Overall tab.			
Missed And Occurred Out Of Window Deviations	The Missed And Occurred Out Of Window Deviations report provides statistics on the Deviation Types and Off Study participants for the protocol.<br><br>The report also provides data on non-deviations, such as occurred visits, planned visits, and Off-Study data, and is provided in this document, rather than as a separate document, to minimize the number of separate reports.	Monthly	The CC Project Manager sends out the Missed And Occurred Out Of Window Deviations Report to everyone who attends the CC-NIDCR-Project Staff Monthly meeting.	Emailed attachment and upload to the UH2/UH3 SharePoint site.<br><br>The Excel report is produced by SAS with data exported from the EDC system.
Non-Visit Timing Deviations	The Non-Visit Timing Deviations report provides statistics on the Deviation Types and Off Study participants for the protocol.	Monthly	The CC Project Manager sends out the Non-Visit Timing Deviations Report to everyone who attends the CC-NIDCR-Project Staff Monthly meeting.	Emailed attachment and upload to the UH2/UH3 SharePoint site.<br><br>The Excel report is produced by SAS with data exported from the EDC system.

Participant-report questionnaire Check	This report provides an accounting of all the questionnaire forms. If there are any errors on any of the forms (in completed, queried, or validated status), they will be listed in this report by Form and also in Summaries by Month, Form, Visit, and Participant.	Monthly	The CC Project Manager sends out the Questionnaire Check Report to the study PI and Project Manager	Emailed attachment and upload to the UH2/UH3 SharePoint site.<br><br>The Excel report is produced by SAS with data exported from the EDC system.
DSMB Report	This contents of this report will be determined by the NIDCR and DSMB members after DSMB is constituted	To be determined by NIDCR	CC creates report draft in collaboration with Co-PIs	Emailed attachment and upload to the UH2/UH3 SharePoint site.<br><br>The Excel report is produced by SAS with data exported from the EDC system.
**Examples of Custom, On-Demand Reports that iSMILE will use for project monitoring**				
Accrual Tracking	This report lists accrual totals stratified by site, language and index versus additional child.<br><br>It also lists the individual participants consented stratified by site and child type (Index v Additional).<br><br>For each ID, the linked ("related" on the report)IDs, consent date, language of consent, off study date, and date entered into REDCAP is provided.<br><br>Accrual totals include both On Study and Off Study participants.<br><br>The report also provides a check of the EDCs to make sure that the information there is complete.	As Needed	Project Staff – can run as needed	Automated report
Accrual Summary By Month And Site	The report lists total accrual stratified by site and month.<br><br>It has distinct sections that include Index children, Cumulative Index children,<br><br>Accrual totals include both on study and off study participants.	As Needed	Project Staff – can run as needed	Automated report

	The report also shows cumulative accrual.			
Participant Contact Listing	This report lists the following information for every study identification number -- Child's Date of Birth, Child's Name, Address, Primary Caregiver's Name, Primary Phone Number, Alternative Phone Number, Email Address, Study Site, and the Consent Language.<br><br>The Participant Contact Listing report also enables searching for the participant ID based on demographic information. Usually the last four digits of the phone that called are used to identify what study ID is linked to that number.<br><br>The report pulls info from participants who are enrolled in the study.<br><br>Participants who have left the study will not show up on this report.	Daily	Project Staff – can run as needed	Automated report
Participants Off Study	The top half of the report provides statistics on the number and proportion of participants Off Study stratified by arm, site, off-study reason, and month.<br><br>The bottom half of the report lists all the participants that have been taken Off-Study and includes the following information for each participant: study ID, Off-Study Date, On Study Date, Days on study, Study Site, Arm, Off-Study Reason, and Explanation.	Monthly	Project Staff – can run as needed	Automated report
Staff Assignments	The Staff Assignments report is used for a variety of purposes, most commonly to see information on staff assignments including study IDs assigned and sites assigned.	As Needed	Project Staff – can run as needed	Automated report
Eligibility Summary	The Eligibility Summary report provides statistics on the questions that make up the Eligibility Questionnaire.	As Needed	Project Staff – can run as needed	Automated report

Enrollment Verification	The Enrollment Verification report checks to make sure participants are eligible for the study and correctly entered.	Bi-weekly	Project Staff – can run as needed	Automated report
Missing Demographics Data By Participant	This report identifies participants that are missing demographics data.<br><br>The following information is provided. The participant's study ID, who the consent was verified by (initials), The date that Consent was signed, The participant's status (e.g. On Treatment).<br><br>Then an 'x' appears in the table under whatever Demographics information is missing for a particular study ID.	Bi-weekly	Project Staff – can run as needed	Automated report
Missing Enrollment Data By Participant	The Missing Enrollment Data By Participant Table report identifies when any of the following enrollment data are missing:<br><br>**Consent:** Consent Form, Signed Date, Version Date, Approved Date, Expiration Date.<br>**Eligibility:** Form Version (this is automatic), Verified By, Status Date, Eligibility Status, eligibility questions<br>**On Study:** Seq. No., On Study Date, Study Site, Subject Staff assignments.<br>Treatment Tab: Step code (automatic), arm code, on arm date.<br><br>If one of the above is missing, the report will list the study ID and list a 'x' in the column for the information that is missing.	Bi-weekly	Project Staff – can run as needed	Automated report
Participant Phone Number Check	The Participant Phone Number Check report checks to make sure the participant child and caregiver's telephone numbers match.	Daily	Project Staff – can run as needed	Automated report
Call Records	The Call Records report is used to identify participants who have not been contacted as our procedures outlined.	Weekly	Project Staff – can run as needed	Automated report
Form Discrepancies	The Form Discrepancies report can be used in two ways.	Weekly	Project Staff – can run as needed	Automated report

	1) The report can be run by the Project Assistant and used to see if they have any pending queries that need to be answered.<br><br>2) The report can be run without checking the box to “show only forms with no response”.<br><br>a) This version of the report includes forms that are in any status but have not yet been validated.<br><br>b) It will also include study IDs that are showing up because a piece of data was entered that was outside the range. Both field and section level queries.			
Staff Visit Schedule	The Staff Visit Schedule report is used by the Project Assistant to find out what visits they have coming up each week.	Weekly	Project Staff – can run as needed	Automated report

Data Closeout and Database lock:

In conjunction with the PIs, the CC will perform continuous data QC during the data collection phase of the project. These processes will identify and ameliorate almost all correctable database errors, on an ongoing basis. In addition, the following closure checks will be implemented:

- Check that the project database and export database are consistent with the specifications in the project data dictionary

- Determine the status of each subject entered (i.e., excluded, completed, withdrawn, lost to follow-up, etc.)

- Check for value formatting problems in database exports

Steps for locking the database:

The PIs will work with the CC and the clinical site monitoring contractor (Rho) to help coordinate site monitoring and study close-out visits and resolve any identified issues, as appropriate.

Following collection of all data, additional data processing will be required, e.g., longitudinal coding of dental examination data, creation of psychosocial scale scores. In conjunction with that data processing, additional QA/QC checks will be made, which may result in identification

of a small number of additional database errors; any such errors will be corrected by the CC data manager, with the assistance of the study team, as appropriate. Following PI concurrence, the database will be locked.

When locking the database, the CC will ensure a full backup is completed and stored on the CC secure drive in a project-specific folder accessible to the CC Directors, or their designated alternate. The CC secure drive is a secure network drive behind the UCSF firewall that requires password-protected, role assignable access, and includes instantaneous local back-up with version control and daily offsite back- up. All study team user rights will be removed from the corresponding EDC system(s). Once the database is locked, access to the export files will be 'read only' and password protected.

An encrypted copy of the final data set will be electronically transmitted to the), The file will be 'read only' and strong password protected.

Statistician or PI(s) confirms the data has been received either via email or faxed receipt, the password will be given via a second e-mail.

16.5 Study Records Retention

All paper documents will be stored in locked secured cabinets at GSDM's research offices at 560 Harrison Avenue in Boston, Massachusetts.

The primary databases will reside on a secured server, accessible to appropriate BU GSDM staff, Agile Health Staff and at the central Data Coordinating Center at UCSF.

All study records and computer files will be retained for at least 3 years after the final financial report for the UH3 award is submitted to the NIH or the final study manuscript is published, whichever is later. At each 3 year interval, the necessity of keeping the study records and computer files longer will be reviewed and decided upon.

16.6 Protocol Deviations

A protocol deviation is any noncompliance with the clinical trial protocol or Good Clinical Practice (GCP) requirements. The noncompliance may be either on the part of the subject, the investigator, or the study site staff. As a result of deviations, corrective actions will be developed and implemented promptly as determined by the IRB.

These practices are consistent with ICH E6:

4.5 Compliance with Protocol, sections 4.5.1, 4.5.2, and 4.5.3

5.1 Quality Assurance and Quality Control, section 5.1.1

5.20 Noncompliance, sections 5.20.1, and 5.20.2.

It is the responsibility of the Principal Investigators to use continuous vigilance to identify and report deviations within 5 working days of identification of the protocol deviation, or within 5 working days of the scheduled protocol-required activity. All deviations must be reported to the BUMC IRB per their guidelines and promptly reported to the NIDCR Program Official. The CC will provide a facility to log protocol deviations. PIs will be responsible for reporting Reportable PDs (including SDs) and UPs directly to their institutional IRB(s) within the specified IRB timeframe

All study staff will have weekly reviews with their supervisor to identify compliance to protocol details. Any and all study related meetings will also dedicate time to review the possibility of identifying any protocol deviations. Protocol deviations will be sent to the BUMC IRB per their guidelines. The PI/study staff will be responsible for knowing and adhering to the IRB requirements.

All deviations from the protocol will be addressed in study subject source documents. A completed copy of the NIDCR Protocol Deviation Form will be maintained in the regulatory file, as well as in the subject's source document.

17 PUBLICATION/DATA SHARING POLICY

This study will comply with the *NIH Public Access Policy*, which ensures that the public has access to the published results of NIH funded research. It requires scientists to submit final peer-reviewed journal manuscripts that arise from NIH funds to the digital archive PubMed Central upon acceptance for publication.

Data from this study will be shared in accordance with the NIH Data Sharing Policy. https://grants.nih.gov/grants/policy/data_sharing/data_sharing_guidance.htm

The National Institutes of Health (NIH) has issued a policy to promote broad and responsible dissemination of information from NIH-funded clinical trials through ClinicalTrials.gov. The policy establishes the expectation that all investigators conducting clinical trials funded in whole or in part by the NIH will ensure that these trials are registered at ClinicalTrials.gov, and that results information of these trials is submitted to ClinicalTrials.gov. Please see https://www.federalregister.gov/documents/2016/09/21/2016-22379/nih-policy-on-the-dissemination-of-nih-funded-clinical-trial-information.

18 LITERATURE REFERENCES

Abroms LC, Ahuja M, Kodl Y, et al. Text2Quit: results from a pilot test of a personalized, interactive mobile health smoking cessation program. Journal of Health Communication. 2012;17 Suppl 1:44-53.

Albino J, Tiwari T, Gansky SA, et al. The basic research factors questionnaire for studying early childhood caries. BMC Oral Health. 2017;17(1):83.

Baron RM, Kenny DA. The moderator-mediator distinction in social psychological research: conceptual, strategic, and statistical considerations. Journal of Social and Personality Psychology. 1986;51(6):173-1182.

Bennett GG, Warner ET, Glasgow RE, et al. Obesity treatment for socioeconomically disadvantaged patients in primary care practice. Archives of Internal Medicine. 2012;172(7):565-574.

Borland R, Balmford J, Benda P. Population-level effects of automated smoking cessation help programs: a randomized controlled trial. Addiction. 2013;108(3):618-628.

Brendryen H, Kraft P. Happy ending: a randomized controlled trial of a digital multi-media smoking cessation intervention. Addiction. 2008;103(3):478-484; discussion 485-476.

Broder HL, Wilson-Genderson M, Sischo L. Reliability and validity testing for the Child Oral Health Impact Profile-Reduced (COHIP-SF 19). Journal of Public Health Dentistry. 2012;72(4):302-312.

Celik S, Cosansu G, Erdogan S, et al. Using mobile phone text messages to improve insulin injection technique and glycaemic control in patients with diabetes mellitus: a multi-centre study in Turkey. Journal of Clinical Nursing. 2014.

Charns MP, Foster MK, Alligood EC, et al. Multilevel interventions: measurement and measures. Journal of National Cancer Institute Monographs. 2012;2012(44):67-77.

Chen YF, Madan J, Welton N, et al. Effectiveness and cost-effectiveness of computer and other electronic aids for smoking cessation: a systematic review and network meta-analysis. Health Technology Assessment. 2012;16(38):1-205, iii-v.

Cohen S, Kamarck T, Mermelstein R. A global measure of perceived stress. Journal of Health and Social Behavior. 1983;24(4):385-396.

Duggan M, Rainie L. Cellphone Activities 2012. Accessed June 6, 2017.

Dye BA, Mitnik GL, Iafolla TJ, Vargas CM. Trends in dental caries in children and adolescents according to poverty status in the United States from 1999 through 2004 and from 2011 through 2014. Journal of the American Dental Association. 2017. *http://dx.doi.org/10.1016/j.adaj.2017.04.013*.

Finitsis DJ, Pellowski JA, Johnson BT. Text message intervention designs to promote adherence to antiretroviral therapy (ART): A meta-analysis of randomized controlled trials. PloS one. 2014;9(2):e88166. doi: 10.1371/journal.pone.0088166.

Fox S, Duggan M. Mobile Health 2012. . http://www.pewinternet.org/2012/11/08/mobile-health-2012/. Accessed on November 15, 2014.

Free C, Knight R, Robertson S, et al. Smoking cessation support delivered via mobile phone text messaging (txt2stop): A single-blind, randomised trial. Lancet. 2011;378(9785):49-55.

Gibbons RD, Hedeker D, DuToit S. Advances in analysis of longitudinal data. Annu Rev Clin Psychol. 2010;6:79-107.

Guerriero C, Cairns J, Roberts I, Rodgers A, Whittaker R, Free C. The cost-effectiveness of smoking cessation support delivered by mobile phone text messaging: Txt2stop. The European Journal of Health Economics. 2013;14(5):789-797.

Hedrick VE, Savla J, Comber DL, et al. Development of a brief questionnaire to assess habitual beverage intake (BEVQ-15): sugar-sweetened beverages and total beverage energy intake. J Acad Nutr Diet. 2012;112(6):840-849.

Herrero J, Meneses J. Short Web-based versions of the perceived stress (PSS) and Center for Epidemiological Studies-Depression (CESD) Scales: a comparison to pencil and paper responses among Internet users. Computers in Human Behavior. 2006;22:830-846.

Hodgson RJ, John B, Abbasi T, et al. Fast screening for alcohol misuse. Addictive Behaviors. 2003;28(8):1453-1463.

Hurling R, Claessen JP, Nicholson J, Schafer F, Tomlin CC, Lowe CF. Automated coaching to help parents increase their children's brushing frequency: an exploratory trial. Community Dental Health. 2013;30(2):88-93.

Kohout FJ, Berkman LF, Evans DA, Cornoni-Huntley J. Two shorter forms of the CES-D (Center for Epidemiological Studies Depression) depression symptoms index. J Aging Health. 1993;5(2):179-193.

Kressin NR, Nunn ME, Singh H, et al. Pediatric clinicians can help reduce rates of early childhood caries: effects of a practice based intervention. Medical care. 2009;47(11):1121-1128.

MacKinnon DP, Fairchild AJ, Fritz MS. Mediation analysis. Annual Review of Psychology. 2007;58:593-614.

MacKinnon DP, Luecken LJ. Statistical analysis for identifying mediating variables in public health dentistry interventions. Journal of Public Health Dentistry. 2011;71 Suppl 1:S37-46.

Marinho VCC, Worthington HV, Walsh T, Clarkson JE. Fluoride varnishes for preventing dental caries in children and adolescents. *Cochrane Database of Systematic Reviews* 2013, Issue 7. Art.No.: CD002279. DOI: 10.1002/14651858.CD002279.pub2.

Mason M, Ola B, Zaharakis N, Zhang J. Text Messaging Interventions for Adolescent and Young Adult Substance Use: a Meta-Analysis. Prev Sci. 2015 Feb;16(2):181-8.

Mauskopf JA, Sullivan SD, Annemans L, et al. Principles of good practice for budget impact analysis: report of the ISPOR Task Force on good research practices--budget impact analysis. Value in Health. 2007;10(5):336-347.

Morris NS, MacLean CD, Chew LD, Littenberg B. The Single Item Literacy Screener: evaluation of a brief instrument to identify limited reading ability. BMC Fam Pract. 2006;7:21.

Palmer CA, Kent R, Jr., Loo CY, et al. Diet and caries-associated bacteria in severe early childhood caries. Journal of Dental Research. 2010;89(11):1224-1229.

Pew Research Center. Mobile Fact Sheet. 2017; http://www.pewinternet.org/fact-sheet/mobile/. Accessed June 8, 2017.

Romano PS, Bloom J, Syme SL. Smoking, social support, and hassles in an urban African-American community. American Journal of Public Health. 1991;81(11):1415-1422.

Saffari M, Ghanizadeh G, Koenig HG. Health education via mobile text messaging for glycemic control in adults with type 2 diabetes: a systematic review and meta-analysis. Prim Care Diabetes. 2014 Dec;8(4):275-85.

Shrout P, Bolger N. Mediation in experimental and nonexperimental studies: new procedures and recommendations. Psychological Methods. 2002;7:422-445.

Siopis G, Chey T, Allman-Farinelli M. A systematic review and meta-analysis of interventions for weight management using text messaging. Journal of Human Nutrition and Dietetics. 2015;28:1-15.

Smith A. Americans and text messaging. 2011; http://www.pewinternet.org/2011/09/19/americans-and-text-messaging/. Accessed February 2, 2015.

Smith PC, Schmidt SM, Allensworth-Davies D, Saitz R. A single-question screening test for drug use in primary care. Archives of Internal Medicine. 2010;170(13):1155-1160.

Stewart JE, Strack S, Graves P. Development of oral hygiene self-efficacy and outcome expectancy questionnaires. Community Dentistry Oral Epidemiology. 1997;25(5):337-342.

Stoyanov SR, Hides L, Kavanagh DJ, Zelenko O, Tjondronegoro D, Mani M. Mobile app rating scale: a new tool for assessing the quality of health mobile apps. JMIR mHealth and uHealth. 2015;3(1):e27. doi: 10.2196/mhealth.3422.

Valeri L, Vanderweele TJ. Mediation analysis allowing for exposure-mediator interactions and causal interpretation: theoretical assumptions and implementation with SAS and SPSS macros. Psychol Methods. 2013;18(2):137-150.

Weinstein P, Harrison R, Benton T. Motivating parents to prevent caries in their young children: one-year findings. Journal of the American Dental Association. 2004;135(6):731-738.

Zickuhr K, Smith A. Digital differences, 2012. http://www.pewinternet.org/2012/04/13/digital-differences/.

SUPPLEMENTAL MATERIALS

None at this time.
