# Supplementary material for: Parent-Targeted Oral Health Text Messaging for Underserved Children Attending Pediatric Clinics: A Randomized Clinical Trial
Source: JAMA Netw Open. 2025 Jan 2;8(1):e2452780. doi: 10.1001/jamanetworkopen.2024.52780 (PMC11696445; doi:10.1001/jamanetworkopen.2024.52780)
Supplement: Supplement 2. — eMethods. Examples of Text Messages by Module eAppendix. COVID-19 Analytic Considerations: Addendum to Study Protocol [file jamanetwopen-e2452780-s002.pdf]

Supplemental Online Content

Borrelli B, Endrighi R, Heeren T, et al. Parent-targeted oral health text messaging for underserved children attending pediatric clinics: a randomized clinical trial. *JAMA Netw Open.* 2025;8(1):e2452780. doi:10.1001/jamanetworkopen.2024.52780

eMethods. Examples of Text Messages by Module

eAppendix. COVID-19 Analytic Considerations: Addendum to Study Protocol

This supplemental material has been provided by the authors to give readers additional information about their work.

© 2025 Borrelli B et al. *JAMA Network Open*.

eMethods. Examples of Text Messages by Module

<u>Oral Health Text Messages (OHT)</u>

Brushing Module:

Need strategies for brushing {c1stname}'s teeth each day? Give stickers after each brushing, sing a song during brushing, and brush together

Visiting the Dentist Module:

Remember to take {c1stname} to the dentist every 6 months! What got in the way? A) too stressed, B) too far, C) I don't have a dentist d) no time e) other

Healthy Eating Module:

Look for foods with less than 10g of sugar per serving. Here's a tool that can help. Search for foods {c1stname} eats: http://bit.ly/2lEaky7

Fluoride:

Q: What does tap water do? Reply 1A) strengthen teeth, 1B) reduce cavity causing bacteria, 1C) repair early-stage cavities, or 1D) all these
If incorrect: The correct answer is all of the above! And tap water costs nothing--zip-zero-zilch!
If correct: Yes, the answer is all of the above! And tap water costs nothing--zip-zero-zilch!

Bottle/Sippy Cup Use

<u>Bottle:</u>

Formula & milk have sugars that stay in {c1stname}'s mouth while she sleeps & can lead to cavities if her teeth are not brushed before bed

<u>Sippy Cup:</u>

If other adults take care of {c1stname}, ask them to only put water in her sippy cup between meals to keep cavities away

Sugary Drinks:

Q: True or False - Grape juice has more sugar than a can of soda. Reply TRUE or FALSE.

The answer is TRUE! Grape juice has 12 teaspoons of sugar, and soda has about 10 teaspoons. Both are high in sugar. Choose water instead!

Bed Time Routine:

Docs recommend 'brush-book-bed': Brush teeth, read a book, & tuck them in. Routines form good habits for kids & eases stress for you!

Fun Facts:

Q: How many quarts of saliva does your mouth produce in a lifetime? Reply 1A = 10,000, 1B = 15,000, 1C = 20,000, or 1D = 25,000

IF 1D You got it right! Your mouth will produce about 25,000 quarts of saliva in your lifetime! That's enough to fill a swimming pool!

© 2025 Borrelli B et al. *JAMA Network Open*.

Child Wellness Text Messages (OHT)

Reading:
Set aside a few quiet minutes every day for sharing books together, perhaps as part of your regular bedtime routine

Safety
Could you find the nationwide poison control center phone number, 18002221222, in 10 seconds or less if you needed it? Reply YES or NO
If YES: Great! Remember, the number is 18002221222, put it on or near every telephone in your home and program it into your cell phone.
If NO: That's OK! The number is 18002221222. Put it on or near every telephone in your home and program it into your cell phone.

Physical Activity
Activity idea! Play freeze dance. Put on your child's favorite music and take turns turning it off and on!

Healthy Development

Does your child go to bed at the same time every night?
If YES: Excellent! The same bedtime every night helps with daytime schedules and a sense of well-being
If NO: It's not easy, but try to build a nightly routine to give your children a sense of security and more self-discipline

Second Hand Smoke:
Kids who are exposed to other's smoke get more chest colds, flu, asthma, ear infections, bronchitis and pneumonia

Stress Tips for You
Stress tip: Deep breathing reduces stress! Try this: Breathe in through your nose, hold 3 seconds, then breathe out through your mouth

Safety Part 2
To prevent burns from fires, BE ALARMED. Install and maintain smoke alarms in your home—on every floor and near every bedroom

Sleep/Behavior/Self Esteem
Behavior Tip: Allow {c1stname} to make simple choices - like choosing socks, foods, or books, to help him feel "in control."

© 2025 Borrelli B et al. *JAMA Network Open*.

eAppendix. COVID-19 Analytic Considerations: Addendum to Study Protocol

Considerations of the iSmile Analytic Plan in response to the impact of the COVID epidemic on the study.

We do not expect the COVID epidemic to have a direct effect on our primary outcome of caries at 24 months, or on secondary outcomes of oral health behaviors. In particular, we would not expect the COVID epidemic to have any differential impact on these outcomes for those receiving the OHT vs. CWT intervention. Also, we do not expect the efficacy of either the OHT or CWT intervention to be impacted by the COVID epidemic. The interventions are administered remotely, and there were no changes to the interventions in response to the COVID epidemic. Therefore, our proposed analytic models will not change in response to the COVID epidemic – primary analyses will not include covariates to model the COVID epidemic or interaction terms between COVID variables and the intervention.

We do expect the COVID epidemic to increase the amount of missing data for our primary outcome measure of any new ECC at 24 months, determined by oral exam (which have been cancelled due to the epidemic). Also, for oral exams that are completed, we expect greater variation in timing of the exams (exams may be performed outside of protocol time windows).

Our protocol states that if follow-up is 70% or greater, primary analyses will be conducted on those with observed data (with possibility of sensitivity analyses to examine the impact of missing data on our analyses). If follow-up falls below the anticipated 70%, the revised protocol states that the primary analysis will account for missing data through multiple imputation. Clarifying that '70% follow-up' means that 70% of randomized participants can be included in the complete case analysis, our protocol does not change with respect to handling missing data (although the likelihood of needing to account for missing data through multiple imputation has increased due to the COVID epidemic).

To include exams performed outside protocol time windows, we will include a 'months from baseline' variable as a covariate in the analyses. This covariate would allow the odds of having new caries to increase for those with later exams. We do not expect that the impact of the timing of the oral exam on study outcomes to have differential effects in the two study groups, and so we do not plan to include interactions between 'months from baseline' and study group.

The following discussion reflects our thoughts about addressing missing data related to the COVID epidemic, and our decision to use multiple imputation as an analytic strategy.

We focus on the primary analysis, using logistic regression to analyze any vs. no new ECC. There are two common approaches to addressing missing data in analysis, through multiple imputation or through inverse probability weighting. Both are valid even with substantial amounts of missing data. Under COVID, those missing oral exams may be close to 50%, but both IPW and multiple imputation should still correct for bias. However, more missing data will lead to a greater loss of power and statistical precision, which will make it harder to show statistical significance. Given the greater potential for missing data due to the COVID pandemic, we revisit the choice of using inverse probability weighting or multiple imputation for the iSmile study.

One preliminary comment is that the primary concern around missing data is bias - those missing data are often different from those with observed data, and ignoring those missing data therefore biases the sample. With COVID, while the amount of missing data may increase, missingness may depend less on subject characteristics (participants are missing oral exams because we stopped conducting exams, independent of a participant's characteristics). That is, during COVID oral exam data may be missing completely at random (MCAR). When data are MCAR, missing data should have less of an impact on bias, and so this increased missingness may not lead to an increase in bias.

© 2025 Borrelli B et al. *JAMA Network Open*.

Both multiple imputation and inverse probability weighting use observe data to address missing data. In inverse probability weighting, observed data are used to model the probability that a participant will be missing the oral exam. The estimated propensity for missing data is then used to create a weight used in analysis of participants with complete data to correct for bias in the followed sample. With COVID, missing the oral exam may not depend on individual characteristics (MCAR), but prior to COVID missing oral exams may have depended on participant characteristics (MAR). This complicates the IPW approach, where weights are based on the propensity for missing data, since the model for this propensity may change. In multiple imputation, observed data are used to model the missing outcome (any new caries). COVID should not impact associations between observed survey data and oral exam data, and so the multiple imputation approach may be more straightforward given COVID.

Given these considerations, and that our revised protocol states that we will use multiple imputation if we need to address missing data, we will stay with the choice of multiple imputation to address missing data.

For secondary outcomes reflecting oral health behaviors over time (at baseline, 4, 12, and 24 months), data will be collected remotely and so it is not clear that the COVID epidemic will increase missing data for these analyses. Analyses of these outcomes will be through random intercept mixed effects regression models, and maximum likelihood estimation of mixed effect regression models appropriately deals with MAR on the dependent variable in repeated measures analyses, provided that there are no missing data on the independent variables in the model. For our analyses of changes in these secondary outcomes over time, where independent variables reflect study group and participant characteristics at baseline, our primary analyses will account for missing data.

© 2025 Borrelli B et al. *JAMA Network Open*.
