# Supplementary material for: Parent-Targeted Oral Health Text Messaging for Underserved Children Attending Pediatric Clinics: A Randomized Clinical Trial
Source: JAMA Netw Open. 2025 Jan 2;8(1):e2452780. doi: 10.1001/jamanetworkopen.2024.52780 (PMC11696445; doi:10.1001/jamanetworkopen.2024.52780)
Supplement: Supplement 3. — Data Sharing Statement [file jamanetwopen-e2452780-s003.pdf]

Data Sharing Statement

Borrelli. Parent-Targeted Oral Health Text Messaging for Underserved Children Attending Pediatric Clinics. *JAMA Netw Open*. Published January 02, 2025.
doi:10.1001/jamanetworkopen.2024.52780

Data
Additional Information: NCT03294590
Data available: Yes
Data types: Deidentified participant data
How to access data: Upon request: belindab@bu.edu or mhenshaw@bu.edu
When available: beginning date: 07-01-2025

Supporting Documents
Document types: None

Additional Information
Who can access the data: Researchers whose proposed use of the data has been approved.
Types of analyses: For a specified research purpose.
Mechanisms of data availability: After approval of a proposal from researchers.
